# The Seed of Industrial Hemp (*Cannabis sativa* L.): Nutritional Quality and Potential Functionality for Human Health and Nutrition

**DOI:** 10.3390/nu12071935

**Published:** 2020-06-29

**Authors:** Barbara Farinon, Romina Molinari, Lara Costantini, Nicolò Merendino

**Affiliations:** Department of Ecological and Biological Sciences (DEB), Tuscia University, Largo dell’Università snc, 01100 Viterbo, Italy; rominamolinari@libero.it (R.M.); lara.cost@libero.it (L.C.)

**Keywords:** THC, *Cannabis sativa* L. legislation, hempseed oil, hempseed proteins, hempseed minerals, antinutritional compounds, phenylpropionammides, bioactive peptides, hempseed dietary supplementation, hempseed-based food

## Abstract

Hempseeds, the edible fruits of the *Cannabis sativa* L. plant, were initially considered a by-product of the hemp technical fibre industry. Nowadays, following the restorationing of the cultivation of *C. sativa* L. plants containing an amount of delta-9-tetrahydrocannabinol (THC) <0.3% or 0.2% (industrial hemp) there is a growing interest for the hempseeds production due to their high nutritional value and functional features. The goal of this review is to examine the scientific literature concerning the nutritional and functional properties of hempseeds. Furthermore, we revised the scientific literature regarding the potential use of hempseeds and their derivatives as a dietary supplement for the prevention and treatment of inflammatory and chronic-degenerative diseases on animal models and humans too. In the first part of the work, we provide information regarding the genetic, biochemical, and legislative aspects of this plant that are, in our opinion essential to understand the difference between “industrial” and “drug-type” hemp. In the final part of the review, the employment of hempseeds by the food industry as livestock feed supplement and as ingredient to enrich or fortify daily foods has also revised. Overall, this review intends to encourage further and comprehensive investigations about the adoption of hempseeds in the functional foods field.

## 1. Introduction

**Figure 1:**
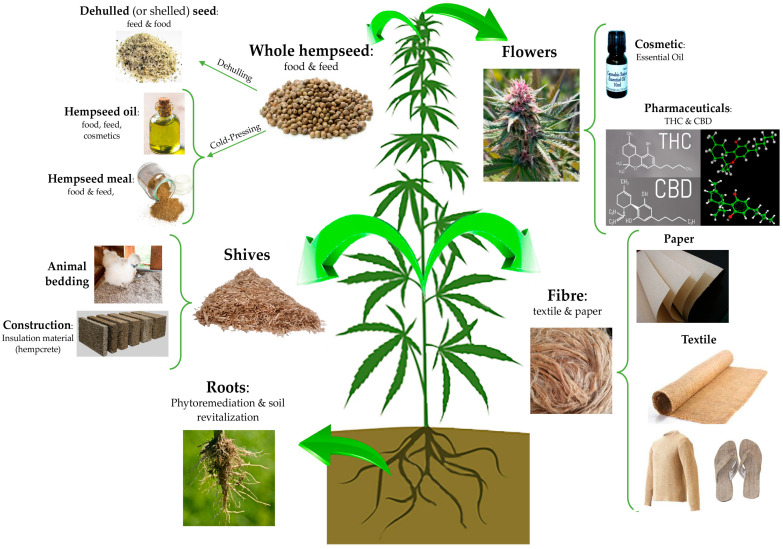
The manifold applications of hemp plant: virtually, each part of this plant can be used in a specific industrial field. The seeds can be used in the food, feed, and cosmetical field as whole or dehulled, or it may be subjected to a cold press process to obtain an oil used in the food and cosmetic industries. From the stem, it is possible to obtain both shives and fibre, useful for animal, building, paper and textile applications. The hemp root system is highly developed in comparison to other herbaceous plants, and this feature is suitable for the phytoremediation of soil from heavy metals. Hemp flowers can be used for ornamental purposes or to obtain products of cosmetic and pharmaceutical interest, such as essential oils composed by delta-9-tetrahydrocannabinol (THC) and cannabidiol (CBD) pure extracts.

*Cannabis sativa* L., commonly known as hemp, is an herbaceous, anemophilous plant belonging to the *Cannabaceae* family. It is considered one of the most ancient cultivated plants and due to its long history of cultivation, it is difficult to identify its exact centre of origin. According to phylogenetic studies based on molecular analysis and studies on sequence homology of ancient and modern DNA extracted from archaeobotanical and modern samples, respectively, most researchers agreed that this plant species originated in central Asia and was introduced in Europe as a cultivated and domesticated agricultural plant during the Bronze age (approximately, from the 22th until 16th century BC) [1,2]. Nevertheless, a recent work by McPartland and colleagues [2] provided evidence that *C. sativa* was indigenous also to Europe. Currently, there are no more traces of wild-type hemp and only domesticated (i.e., individuals of a species chosen and selected by humans for characteristics making them useful to people) and ruderal (i.e., forms growing outside of cultivation) hemp plants exist. Independently to its origin, the nowadays-domesticated form of *C. sativa* L. is widespread and cultivated not only in the Asian countries, but also in Canada, the United States (US), Europe, and Africa. It is a multipurpose, sustainable, and low environmental impact crop which can be useful for several application fields, from the agricultural and phytoremediation to food and feed, cosmetic, building, and pharmaceutical industries. Indeed, from this highly versatile plant, it is possible to obtain various products of industrial interest such as fibre and shives; bio-building and thermal insulated materials; seeds, flour and oil with important nutritional and functional features; and bioactive compounds of pharmacological interest [3] (Figure 1).

Essentially, *C. sativa* L. can be grown for three main purposes: industrial, narcotic/recreational, and medicinal [4]. Traditionally, *C. sativa* L. plants were cultivated primarily as a fibre crop for the production of textiles and ropes, especially in the western world. Despite their high nutritional value, the seeds of this plant were initially considered as a by-product of the fibre production, and hence, they were mainly used as animal feed. From the first half of the 21st century, the cultivation of this crop declined because of the progressive diffusion of synthetic fibres and the use of some narcotic strains of the *C. sativa* L. plant for the production of intoxicant drugs. Only since the last two decades, there has been a reintroduction of the the *C. sativa* L. cultivation exclusively for industrial purposes, and in this context, Canada has been the first western country to restore this crop, followed by Europe and the US. Nowadays, a growing interest for the seeds of the *C. sativa* L. plant, commonly named hempseeds, has been developed due to the increased knowledge about their high nutritional value and potential functionality. However, there is still a lack of awareness and much confusion between “industrial hemp” and “drug hemp” especially among the public opinion, so the research of the potential health benefits provided by hempseed is still penalized by the negative reputation of drug hemp, which daunts interests and investments. Moreover, although in scientific literature there is a fairly good amount of information about the nutritional composition of hempseeds, there is still a lack of studies regarding the nutritional and health benefits related to hempseed-based food products for human consumption. Hence, in this review, we overview the literature about the nutritional and functional properties of hempseeds belonging to those varieties of *C. sativa* L. that have been allowed for cultivation. These varieties contain a delta-9-tetrahydrocannabinol (THC) level less than 0.3% or 0.2% of the reproductive part of the female plant at flowering and are named “industrial hemp”. Next, the literature studies about animal and human hempseed supplementation is reviewed. For more clarity and awareness, in the first part of the work, we provide information regarding the main biochemical, genetic, and legislative aspects of the *C. sativa* L. species, important to outline and understand the difference between industrial hemp and drug hemp.

## 2. Biochemistry, Genetic, Taxonomy and Legal Status of *C. sativa* L.

### 2.1. The Cannabinoids Synthesis in C. sativa L.

The major discriminant factor related to the different intended uses of *C. sativa* L. is the level of the two major and more known phytochemicals characteristic of this crop, namely the only one psychoactive and toxicant compound of the plant, THC, and the non-psychoactive cannabidiol (CBD). Both of them belong to the cannabinoids’ class which includes over of 100 secondary metabolites belonging to the family of terpenophenolic compounds, typical of all *C. sativa* L. plants. These compounds are synthesized, collected, and stored in stalked glandular trichomes, that are specialized tiny secretory epidermal glands [5,6], which are essentially present and abundant on the inflorescence of the female plant, whilst are present in lower numbers on leaves and stems, and are absent on roots and seeds, therefore, these latter organs do not contain cannabinoids [4,7,8]. A possible presence of cannabinoids in hempseeds could occur during the harvesting process, as a result of physical contact with the resin secreted by the glandular trichomes located on the bracts that surround the seed [9,10]. Hence, the presence of cannabinoids in hempseed actually represents a contamination, and the level of this contamination depends on both the *cultivar* (*cv*) and the cleaning process of the seed. Reasonably, THC contamination in seeds from *C. sativa* L. varieties which produce a low-THC level—as the industrial hemp varieties—should be extremely low [9]; anyway, the adoption of a method for the quantification of the possible cannabinoid’s contamination and the level in hempseed products and food may be appropriate [11,12,13].

*C. sativa* L. plants grown for an industrial purpose, are cultivated to obtain fibre, seeds, and their derivatives. These plants are popularly called “industrial hemp” or “fibre-type” hemp, and they contain low-THC level (i.e., <0.3 or 0.2%), whereas, *C. sativa* L. plants cultivated for narcotic/recreational purposes are characterized by high-THC level and those cultivated for medicinal purposes are characterized by high-THC and high-CBD levels.

**Figure 2:**
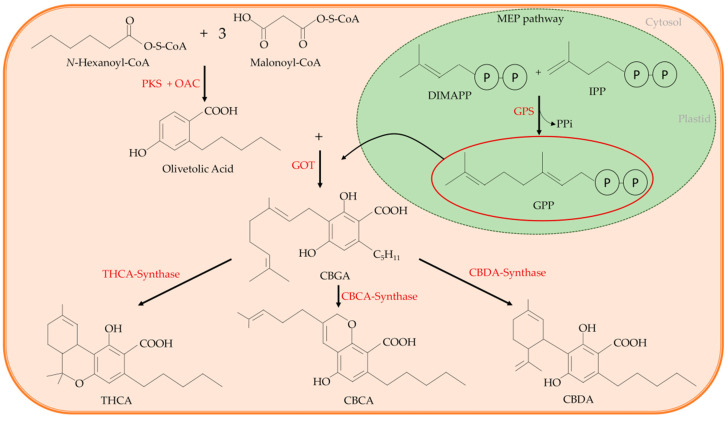
The cannabinoid synthetic pathway: cannabigerolic acid (CBGA) is the common precursor of all main cannabinoids. It is synthesized through an alkylation of the phenolic moiety of olivetolic acid with the terpenoid component of geranyl pyrophosphate (GPP). The reaction is catalysed by a geranylpyrophosphate:olivetolate geranyltransferase (GOT). Olivetolic acid is originated in the cytosolic polyketide pathway through an aldol condensation of hexanoyl-Coenzyme A (CoA) with three molecules of malonyl-CoA, that is catalysed by the polyketide synthase (PKS) enzyme in the presence of olivetolic acid cyclase (OAC). The GPP is synthesized by the plastidial methylerythritol phosphate (MEP) pathway. In the cytosol, CBGA is converted into the acidic form of the three main cannabinoids, tetrahydrocannabinol acid (THCA) that in the acidic form has no psychoactive activity, cannabidiolic acid (CBDA) and cannabichromenic acid (CBCA). GPS: geranyl pyrophosphate synthase; IPP: isopentenyl diphosphate; OAC: olivetolic acid cyclase.

Several works clarified well the cannabinoids’ biosynthetic pathway [14,15,16,17,18,19]. According to these studies, a common precursor of all the main cannabinoids exists, and it is the cannabigerolic acid (CBGA). In the cytosol, CBGA is converted into the acidic form of the three main cannabinoids, from which other related cannabinoid compounds will originate, namely tetrahydrocannabinol acid (THCA), that in the acidic form has no psychoactive activity; cannabidiolic acid (CBDA); and cannabichromenic acid (CBCA). This conversion is catalysed by an oxidocyclase specific for each cannabinoid (THCA-synthase, CBDA-synthase, and CBCA-synthase, respectively) (Figure 2). Finally, the acidic form of each cannabinoid undergoes non-enzymatic decarboxylation to their neutral and active form, i.e., THC with psychoactive activity, CBD, and CBC that is found at high levels in juvenile plants [20,21], respectively.

### 2.2. Chemical Phenotype and Taxonomy of C. sativa L.

Elucidation of the cannabinoids’ biosynthetic pathway has been essential to demonstrate that the concentration of each cannabinoids in the plant is genetically determined, so that various genotypes related to different chemical phenotypes, diverging in types and concentration of cannabinoids (i.e., cannabinoids profile), exist. These phenotypes are known as “chemotypes” or “biotypes”, and three different principal chemotypes were commonly identified [8,20,22] on the basis of the two main cannabinoids (i.e., THC and CBD) content and ratio:

- Chemotype I is characterized by a low CBD/THC ratio (0.00–0.05), due to high THC content (>0.3% of dry weight of the reproductive part of the female plant at flowering). This chemical phenotype is also known as “drug type”, “THC-predominant”, or *C. sativa* L. subsp. *Indica* and the varieties belonging to this chemotype are those commonly grown for narcotic/recreational purposes.
- Chemotype II has both the two main cannabinoids, CBD and THC, in a content ratio (CBD/THC) close to the unity (0.5–3.0), usually with a slight prevalence of CBD. This chemical phenotype is also named “intermediate type” or “THC-intermediate”, and the varieties belonging to this chemotype are mainly grown for medicinal use.
- Chemotype III is characterized by high CBD/THC ratio (15–25) due to high CBD amount and low THC content, not over than 0.3% of dry weight of the reproductive part of the female plant at flowering. This chemical phenotype is also known as “non-drug type”, “fibre-type”, “THC-predominant”, or *C. sativa* L. subsp. *Sativa*, and the varieties belonging to this chemotype are cultivated for industrial purposes, namely, for fibre, seeds, and their derivatives.

De Meijer and colleagues [23] for the first time gave a clear genetic meaning to the tripartite distribution of the chemotypes within the *C. sativa* L. population. Indeed, studying the inheritance of the chemotype traits of *C. sativa* L. plants, they identified the existence of a single locus named *B* locus, with two co-dominant alleles, named *B_T_* and *B_D_*, each of which codes for the THCA- and CBDA-synthases, respectively. Hence, according to this model, the THC-predominant phenotype (chemotype I) is related to the *B_T_*/*B_T_* genotype, the CBD-predominant phenotype (chemotype III) is determined by the *B_D_*/*B_D_* genotype, and the intermediate phenotype (chemotype II) is induced by the heterozygous state *B_T_*/*B_D_*. The same authors also showed that the value of the CBD/THC ratio in the heterozygous hybrids obtained from cross between parental homozygous pure-THC and pure-CBD, unexpectedly differed significantly and deviated from the expected 1.0 value. Therefore, they speculated that some heritable factor could affect the balance between THCA- and CBDA-synthase in their competition to convert the CBGA precursor. In particular, the authors hypothesized that the *B_T_* and *B_D_* alleles could be part of a wider allelic series coding for several isoenzymatic forms of THCA- and CBDA-synthase, respectively, with differential affinities for the CBGA substrate, resulting in significantly different CBD/THC ratios observed in the heterozygotes. Further studies pointed out the existence of several allelic variants of *B_D_* [7,20,24] and *B_T_* [7,8,24,25] genes coding for less or totally non-functional CBDA- and THCA-synthase, respectively, and which therefore, influence the cannabinoid profile of the plant and, from a practical point of view, also represent an useful genetic marker to differentiate the drug-type from the fibre-type *C. sativa* L. plants. Interestingly, a higher number of allelic variants of wild-type *B_D_* locus was found in comparison to the *B_T_* locus. Hence, considering the high number of the cannabinoid synthase genes’ allelic variants and the higher mutation rate of the CBDA-synthase allele, Onofri and colleagues [7] proposed a phylogenetic hypothesis according to which it is possible to consider all cannabinoids’ allelic variants as a gene family and to speculate that the wild-type CBDA-synthase allele may be the ancestral form of this gene family, from which events of duplication would have led to a higher CBDA-synthase variation, resulting in the formation of the CBDA-synthase pseudogenes (i.e., the CBDA-synthase allelic variants) and to the rise of a new sequence coding for a new enzyme able to convert the CBDA substrate in a new product, the THCA. According to this evolutionary theory, it has also been hypothesized that the low-functionally THCA-synthase allele found in some fibre type (CBD-predominant) plants [25], could be an evolutionarily intermediate between the CBDA-synthase ancestor and the fully functional THCA-synthase allele.

The high intrinsic genetic variability rate of *C. sativa* L. has been further accentuated by the long history of its domestication. Indeed, the different intended uses of the *C. sativa* L. cultivation’s products have led over the years, to an artificial phenotypic selection of specific features of the domesticated plants, useful for increasing the yield and/or the quality of the commercial interest’s cultivation products [26]. The direct consequence of this selection was the unaware artificial creation of the *C. sativa* L. varieties, each with specific genotypic and phenotypic features, which at first, induced the taxonomists and botanists to erroneously recognize two or three different species of *C. sativa* L., embracing a polytypic concept of the *Cannabis* genus [27]. To further complicate the taxonomic classification of the *Cannabis* genus, there has been also the fact that *C. sativa* L. is a crop which tends to exist in “crop-weed complexes”, that is complexes of domesticated forms in cultivation and related ruderal (weedy) forms growing outside of cultivation, developing morphological characteristics also very different from those of the domestic progenitor, as a consequence of adaptation to the wild environment [28]. However, it must be considered that, despite the high genetic variability of *C. sativa* L., the varieties that genotypically and phenotypically differ, are interfertile. Therefore, taking into account the Darwinian definition of biological species, “a group of organisms that can reproduce with one another in nature and produce fertile offspring”, *C. sativa* L. varieties cannot be consider as different species of the *Cannabis* genus. For this reason, to date, the polytypic concept has been definitely given up and replaced by the monotypic one. According to this, a single species of *Cannabis* genus exists, namely *C. sativa* L., which includes several varieties or *cultivars* (*cvs*) that genotypically and phenotypically differ, but they all are interfertile and therefore, they belong to the same species [29,30].

From a practical point of view, the main discrimination factor among the *C. sativa* L. *cvs* is the THC content, which, essentially for legal reasons, as described in the next paragraph, is an useful tool to discriminate between the drug-type plants with high THC content, used for medical or recreational purposes, and the fibre-type plants with low THC content, commonly named industrial hemp.

### 2.3. Legislation of C. sativa L.

Historically, industrial hemp or simply, hemp, that is, *C. sativa* L. plants grown for fibre and/or seeds, was frequently cultivated over the world, mainly for the production of technical textiles, until the first half of the 21st century. In the US, hemp was widely grown from the colonial period into the mid-1800s. In the early 1900s and prior to the late 1950s, hemp continued to be grown, being considered as an agricultural commodity: the US Department of Agriculture (USDA) supported its production, and USDA researchers continued to publish information related to hemp production and also reported on hemp’s potential for use in textiles and in paper manufacturing [31]. In Europe, at the end of the 1950s, Italy was the second country in the world after Russia for the areas under hemp cultivation (over 100,000 hectares) and was the world’s best for the quality of the obtained products [32]. However, following the discovery of the psychotropic activity of THC, and the increasing awareness of its deleterious effects on human health, many countries began to take measures in an effort to stem the use of *C. sativa* L. plants’ flowers and leaves for their psychotropic effects. The first provision was taken in the US and Canada. In the US, between 1914 and 1933, 33 states passed laws restricting legal production to medicinal and industrial purposes only. In 1937, the Marihuana Tax Act defined hemp as a narcotic drug, without any distinguishing between low THC plants (hemp) and high THC (drug hemp or simply, marijuana) ones: both were considered schedule I controlled substances, and it was required that farmers growing hemp hold a federal registration and special tax stamp. This effectively limited further production expansion; in fact, after 1943, production of hemp started to decline until the late 1950s when no production was recorded. Finally, in 1970, The Controlled Substances Act (CSA) was issued, and it placed the control of selected plants, drugs, and chemical substances under federal jurisdiction. Among the selected plants, there were also *C. sativa* L. ones to which were given the statutory definition of marijuana and were put in the Drug Enforcement Administration (DEA) schedule of controlled substances [31]. In Canada, the cultivation of hemp has been prohibited due to the presence of THC, in 1938 with the Canadian Opium and Narcotics Act [33,34]. In 1961, the United Nation (UN) endorsed and adopted the single convention on narcotic drugs, which established a universal system for limiting the cultivation, production, distribution, trade, possession, and use of narcotic substances to medical and scientific purposes, with a special focus on plant-derived substances, among which is cannabis. In the article 28, paragraph 2 of this convention, cannabis was defined as “the flowering or fruiting tops of the *C. sativa* L. plant (excluding the seeds and leaves when not accompanied by the tops) from which the resin has not been extracted, by whatever name they may be designated”. The same article described a system of control required if a country decides to permit the cultivation of *C. sativa* L. that is not for industrial or horticultural purposes [4,35]. Ten years later, in 1971, the UN endorsed the convention on psychotropic substances which established an international control system for psychotropic substances, among which is THC [36]. In line with these directives, in 1975 the Italian Republic issued the law n. 685/1975, introducing cannabis (intended as a drug product obtained from *C. sativa* L. plants) in the schedule of controlled substances.

The better knowledge of the biochemical and biomolecular features of the *C. sativa* L. species has made it possible to understand the genetics and biochemical mechanisms previously described, which are the basis of the cannabinoids’ synthesis and, in particular, of THC. Furthermore, thanks to the development of specific analysis techniques (e.g., gas chromatography or gas chromatography-mass spectrometry), it is now possible to resolutely and accurately quantify the THC content of *C. sativa* L. plants in order to distinguish between *cvs* with high and low THC contents. For these reasons, nowadays, the cultivation of industrial hemp has been reintroduced either in the US, Canada, and Europe. Canada was one of the first country to restore industrial hemp cultivation. Indeed, in 1994, it began to issue licenses for hemp as a research crop and then, in 1998, the cultivation of hemp varieties containing less than 0.3% THC of the dry weight of leaves and flowering parts was legalized, and it is currently permitted, provided that a license from the Office of Controlled Substances of Health Canada has been acquired. To date, Canada is the major hemp-producing and -exporting country, particularly of hemp-based foods, ingredients, and other related products [31]. In the EU, the hemp cultivation reintroduction took place in 2013 with the EU regulation n. 1307/2013 that allowed the growth of *C. sativa* L. plants for industrial purposes only for those plants with low levels of THC. According to this regulation, the granting of payments under the Common Agricultural Policy (CAP) is conditional upon the use of certified seeds of specific hemp varieties, that is, *C. sativa* L. *cvs* with a THC content not exceeding 0.2% of the dry weight of leaves and flowering parts [4]. Thus, the EU has adopted more stringent parameters compared to Canada, to ensure the safety of and to protect the health of citizens. Different genotypes of industrial hemp with a THC content < 0.2% have been selected and registered, and currently, there are about 70 allowed industrial hemp varieties listed in the European Plant Variety Database as agricultural species (Table 1) [37,38]. Some of these *cvs* are dioecious as the *C. sativa* L. plant naturally occurs; other *cvs* are monoecious and are obtained by ancestral breeding [39]. Often, but not always, the monoecious varieties are adopted for seed production since they give a higher yield of the product of interest, whereas the dioecious *cvs* are mainly adopted for fibre production. Moreover, the industrial hemp varieties listed in the EU plant variety database are constantly updated based on the results of the annual THC-content’s monitoring and on the possible request for the introduction of new *cvs* with a low THC amount.

Nowadays the EU is the world’s largest hemp-producing market second only to Canada, with France, the Netherlands, Lithuania, and Romania as the major production centres [31]. According to the EU guidelines, also in Italy, the cultivation of industrial hemp has been recently restored through the law n. 242/2016, and the subsequent circular of the Ministry of Agricultural, Food and Forestry Policies (MIPAAF) published on 14 January 2017 that has delineated the conditions for hemp production, its commercialization, and its utilization for specific industrial purposes [38].

**Figure 3:**
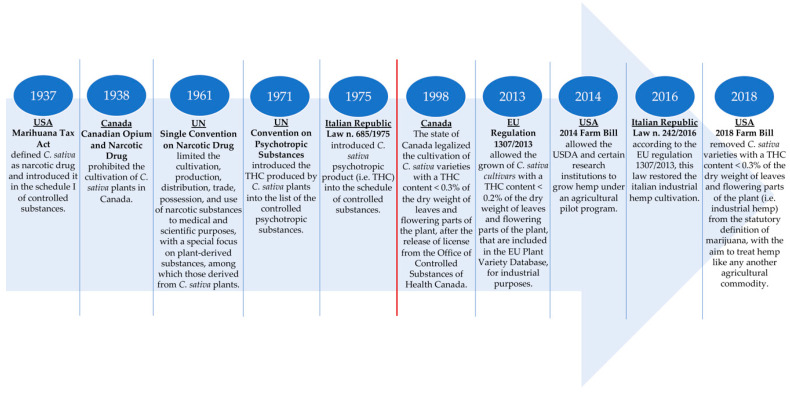
The main points of the legislation about *C. sativa* L. (industrial hemp) cultivation in the US, Canada, and the EU with a focus on the Italian Republic among the EU states. The red line separates the legislation related to the prohibition of *C. sativa* L. cultivation (left part of the red line) from that allowed the reintroduction of growing of this crop (right part of the red line). For more details, see Section 2.3. US: United States; UN: United Nations; THC: delta-9-tetrahydrocannabinol; EU: European Union; USDA: United Stated Department of Agricultural.

Finally, in the US, the federal policy regarding hemp was significantly altered with the 2014 Farm Bill (Agricultural Act of 2014) that allowed the USDA and certain research institutions to grow hemp under an agricultural pilot program. Despite this act, industrial hemp continued to be a niche crop. The great novelty was four years later with the 2018 Farm Bill that has established a new federal hemp regulatory system under the USDA with the aim to facilitate the commercial cultivation, processing, and marketing of hemp and to essentially treat hemp like any other agricultural commodity. Indeed, it removed hemp (i.e., *C. sativa* L. varieties with a THC content <0.3% of the dry weight of leaves and flowering parts) and their products—among which is hempseed—from the statutory definition of the drug marijuana and the DEA schedule of controlled substances, opening the hemp industry for business [31]. 2018 was also the year in which the federal government of Canada legalized access to recreational cannabis (i.e., the drug-type *C. sativa* L.) through the entry into force of the *Cannabis Act*, Bill C-45 [40]. In Figure 3, the main highlights about the *C. sativa* L. legislation in Canada, the US, the EU and Italy among the EU states, are illustrated.

## 3. *C. sativa* L. Seed

As mentioned above, the *C. sativa* L. crop has a prehistoric use as an important source of industrial interest’s plant fibre. Nevertheless, in the last decades, there has been a growing interest in the seed of the plant which is the fruit of hemp, commonly named seed, even if it is technically an achene, namely an one-seeded dry fruits in which the pericarp is not as tightly joined to the seed, essentially similar to the cereal caryopsis [28]. Normally, the seeds of hemp grown for fibre production were considered as a waste product, and at most, they were mainly used as animal feed [34,41]. However, in recent times, with the growing recognition of their nutritional features and health benefits, the production of hempseeds has been increased and these seeds have become a product with an important and growing potential market [42]. Therefore, the literature about the nutritional and functional features related to this product has been reviewed in the next sections.

### 3.1. Hempseed Nutritional Features

Hempseed has commonly been claimed as one of the most nutritionally complete food sources due to its high nutritive traits. It can be consumed as such (whole, hulled seed) or dehulled (hempseed kernel), as well as its processing products, including oil, flour, and protein powder. Despite the fact that some studies highlighted high variability in the hempseed composition according to the genotypes and environmental factors [3,32,34,39,43,44,45,46], it typically contains 25–35% lipids with a unique and perfectly balanced fatty acids (FAs) composition; 20–25% proteins easy to digest and rich in essential amino acids; 20–30% carbohydrates, a great part of which are constituted in dietary fibre, mainly insoluble; as well as vitamins, and minerals (Table 2).

In addition to its nutritional value, hempseed is also rich in natural antioxidants and other bioactive components such as bioactive peptides, phenolic compounds, tocopherols, carotenoids, and phytosterols, the content of which appears to be mostly affected by the environmental and agronomic factors and, to a lesser extent, by genetic variability [3]. Moreover, hempseed contains also some antinutritional compounds that could negatively influence its nutritional value. In the following subsections, these nutritional and antinutritional hempseed’s components are discussed in detail.

#### 3.1.1. Hempseed Fat

##### Fatty Acid Composition

Fat represents the most important component of hempseeds, particularly from an industrial point of view. Indeed, since hempseeds are oilseeds, the oil is the main food product of industrial interest that it is possible to obtain from them. For this reason, hempseed’s fat is commonly called oil. Several studies [34,39,44,45,46,47,48,49] demonstrated that the oil content of hempseeds belonging to different *cvs* ranges from 25 to 35% of the whole seed (Table 2). Galasso and colleagues [39] and Irakly and co-workers [3], analysing the seeds belonging to different industrial hemp *cvs*, highlighted that this variation is mainly due to the genotype. In addition, Irakli and colleagues [3] also observed that even environmental conditions such as geography, climatic conditions, and local agronomic factors have an effect on the total oil content, though slighter than genotype. This evidence is in accordance with the findings of Kriese and colleagues [41] and Mihoc and co-workers [43]. In this latter study, the authors, analysing the oil content of five Romanian industrial hemp *cvs* grown in two consecutive years in the same site, found that environmental conditions such as high monthly average temperature of 25–26 °C and a low rainfall level (223 mm during the flourishing of plants) resulted in an incomplete maturation of hempseeds and in a decrease in oil content.

As regards the chemical composition of the hempseed’s fat, it is needed to consider that most publications on this argument are referred to the oil extracted from hempseed through specific industrial methods, rather than to the whole seed, because of the high industrial relevance attributable to hempseed oil. However, in this review, we have been focused primarily on the whole hempseed, so, except in the case of a lack of literature data about whole hempseed, we have considered only the publications about this matrix, excluding those specifically concerning the oil. The literature data about the FA composition of the fat component of whole hempseed are reported in Table 3.

Overall, the literature data showed that hempseed oil is characterized by high polyunsaturated fatty acids (PUFAs) content and low saturated fatty acids (SFAs) amounts. More precisely, based on genotype and environmental factors, hempseed oil contained up to 90% unsaturated fatty acids [34], of which from 70% to over 80% is composed by PUFAs. The major monounsaturated fatty acid (MUFA) was Oleic Acid (18:1, n-9, OA) which has been shown to reach the highest value (18.78%) in the Canadian *cv* Joey [44], whereas the lowest OA content (8.42%) was found in the Finola *cv* grown in Italy [39]. Generally, the amount of OA in hempseed oil is shown to be higher than those found in chia seed (7%) [51] and comparable to those present in linseed (15%) [52]. Among PUFAs, Linoleic Acid (18:2, n-6, LA) was the most representative FA in hempseed oil of all analysed genotypes, accounting for more than half of the total FA. The second prominent PUFAs was α-linolenic acid (18:3, n-3, ALA). Hence, hempseed oil represents an especially rich source of these two fatty acids which are known as Essential Fatty Acids (EFAs), since they cannot be synthesized by mammals and, therefore, must be acquired by diet because they are necessary to maintain healthy human life. Indeed, LA and ALA are the precursors of the n-6 and n-3 PUFAs biologically active in animals, including humans, namely the long-chain PUFAs Arachidonic Acid (20:4, n-6, AA) which derived by the conversion of LA; and Docosahexaenoic Acid (22:6, n-3, DHA) and Eicosapentaenoic Acid (20:5, n-3, EPA) obtained from the ALA precursor. These biologically active forms of EFAs are necessary for many physiological processes, including maintenance of cell membrane structure, and cardiovascular health, the regulation of metabolic and inflammatory processes through the synthesis of prostaglandins and leukotrienes, skin integrity, as well as proper regulation of the brain’s development and function. In this context, it is important to note that the conversion of both LA and ALA into their biologically active PUFAs derivatives occurs at a low conversion rate. Several studies [3,34,39,47] highlighted that the Finola *cv* was the hemp variety with the highest ALA amount compared to other *cvs*. The highest ALA content in the Finola *cv* was found by Callaway (22%) [47] and the lowest was found by Irakli and colleagues (15.3%) [3].

In addition to the absolute concentration of PUFAs in the diet, the n-6/n-3 ratio represents an important index to ensure the maintenance of an optimal state of health, and to prevent the onset of chronic-degenerative diseases characterized by a chronic inflammation status, like cardiovascular and neurodegenerative diseases, as well as cancer [53]. Despite the existence of different lines of thought about the healthiest n-6/n-3 ratio, according to the European Food and Safety Authority (EFSA) [54], the ideal n-6/n-3 ratio has been established as 3:1 to 5:1, that is also the ratio found in the traditional Japanese and Mediterranean diets, where the incidence of coronary disease has been historically low [47]. The levels of LA and ALA found in the fat component of hempseeds make them a food matrix with the ideal, low n-6/n-3 ratio ranging precisely from 3:1 to 5:1, which is useful for reducing the n-6/n-3 ratio in diets, especially of industrialized countries, where there is an unhealthy average n-6/n-3 ratio of about 10:1, due to the too high n-6 and too low n-3 amount in the people’s diets [49]. In addition to LA and ALA, among n-6 and n-3 FAs, hempseed oil also contains their respective biologic metabolites γ-Linolenic Acid (18:3, n-6, GLA) and Stearidonic Acid (18:4, n-3, SDA) that allow to bypass the first critical enzymatic step of δ-6-desaturase, facilitating the conversion into the biologically active form of long-chain PUFAs. Even in this case, Finola *cv* appeared as the hemp variety with the highest content of both GLA and SDA [3,34,39,47]. Furthermore, GLA performs also an anti-inflammatory activity since it is rapidly converted in Dihomo-γ-Linolenic Acid (DGLA; 20:3, n-6) in the human body. DGLA is located in the cell membrane, where it can act as a precursor of anti-inflammatory metabolites and can compete with AA for the synthesis of metabolites involving in the inflammatory response [55]. In this context, it is important to consider that hempseed oil is not the only one source of GLA, neither the plant source with the highest amount of GLA, in fact, one of the most representative sources of this FA is the borage oil (19–23% of GLA) which however lacks n-3 PUFAs differently from hempseed oil [56]. As regard the SFAs, studies reported that the total amount of them is not over than 12% (Table 3), with a consequent recommended high (>10) PUFA/SFA ratio, considered beneficial to reduce the risk of atherosclerosis and coronary heart disease [32]. The principal SFA was the Palmitic Acid (16:0, PA) with an amount ranging from 2 to 9%, followed by Stearic Acid (18:0, SA).

Concerning the variability in the hempseed oil’s FAs content, in literature there are some conflicting data. Particularly, a common evidence is that the FAs composition differs among *cvs*. Irakli and co-workers [3] demonstrated that, similarly to the oil content, the FAs profile of hempseeds is drastically affected by genotype. The same authors found that the content of FAs of seven industrial hemp *cvs* approved for cultivation in Greece and grown in the same site for three consecutive years, was most affected by genotype for ALA primarily, followed by OA and PA (genotype contribution on the variance, 99.6%, 91.2%, and 86.2%, respectively), whereas LA was the FA least affected (genotype contribution on the variance, 42%). Similar data were found by Vonapartis and colleagues [34] for ten Canadian industrial hemp *cvs*, which did not obtain significant differences among the analysed *cvs* for LA, but they found noticeable differences in the ALA concentration. Contrarily, Galasso et al. [39] detected high and significant variability for both LA and ALA contents of twenty hempseed accessions and *cvs*.

##### The Unsaponifiable Matter

Generally, any oil matrix is composed of saponifiable and unsaponifiable matter. The former consists of all the oil substances containing an ester portion that can be removed by treatment with irreversible alkaline hydrolysis, including mono-, di-, and tri-acylglicerols and phospholipids. The latter, according to the International Organization for Standardization (ISO) 18609:2000 definition, consists in all the substances dissolved in the product which cannot be saponified by the caustic alkalis but are soluble in the ordinary fat solvent (e.g., hexane). The unsaponifiable matter includes sterols, aliphatic and terpenic alchols, as well as fat-soluble vitamins. Literature data about the composition of the unsaponifiable matter of the fat component of whole hempseed are very scarce; in fact, almost all of them concern the oil of hempseed. One of the first reports in which the unsaponifiable matter of the oil of hempseed has been investigated in detail was the work of Montserrat-De La Paz and colleagues [57]. They demonstrated that the unsaponifiable matter of hempseed oil represents from 1.8 to 1.92% of the total oil, and the most relevant compounds of this oil fraction are tocopherols and phytosterols. Tocopherols are naturally occurring, fat-soluble compounds with high antioxidant activity. They include the different isomers α-, β-, γ-, and δ-tocopherol and represent the dominant antioxidants in hempseed oil, protecting it from oxidation thanks to their ability to scavenge free radicals. As said, most publications in which the tocopherol content has been assessed concern the oil of hempseed instead of whole hempseed, and in fact, the obtained results are expressed referring to the oil (e.g., mg/100 g of oil) rather than to the seed (e.g., mg/100 g of seeds). Anyway, they provide important information about the content of these functional compounds, and considering the lack of data referred to whole hempseed, these publications have been considered in this specific case. The literature data obtained from the studies that evaluated the tocopherol profile of hempseed and hempseed oil are reported in Table 4, where the results expressed per 100 g of seeds have been separated from those expressed per 100 g of oil.

Obviously, when the values are reported per 100 g of oil, the tocopherol amounts are higher in comparison to the values referring to 100 g of seeds, as a consequence of the concentration of these compounds in the extracted oil. In any case, all works reported γ-tocopherol as the most abundant isomer, followed by α-, δ- and β-tocopherols. Among these isomers, γ-tocopherol has been suggested to be the most active antioxidant in lipids [32], and therefore, it contributes together with other antioxidant compounds like polyphenols to provide high oxidative stability to hempseed and, especially, to its oil, whereas, the α-isoform is considered as the only one tocopherol bioactive form, namely the form with vitamin E activity in the human body [62]. Regarding the daily intake of vitamin E as α-tocopherol, EFSA has set an average intake (AI) of 13 mg/day for males and 11 mg/day for females [62]. According to literature data (Table 4), the α-tocopherol content in whole hempseed can reach up to 5 mg/100 g of seeds [47]. A serving portion of hempseed corresponds to 30 g [44]; hence, the daily intake of a serving portion of whole hempseed can contribute to satisfying up to 14% for women and 12% for men for the vitamin E AI, whereas, the γ-tocopherol content found in the oil of hempseed has been shown to be higher than those found in both linseed and canola seed oils [52].

The total tocopherol amount in hempseed oil has been shown to be higher than that found in sunflower, sesame, and amaranth oil [48]. It can reach even values higher than 90 mg/100 g of oil based on the type of extraction process used to obtain oil from the seed. Indeed, on an industrial scale, various oil extraction techniques exist, each of which can differ in performance in terms of economic costs, extraction yield, and composition and physicochemical quality of the extracted oil [63]. One of these techniques is the cold-pressing method which, however, has low extraction efficiency and leads to obtaining an oil with high chlorophyll levels, probably due to intensive mechanical destruction of hempseed cells during the pressing and the consequent release of the pigments from chloroplasts. High amounts of chlorophyll negatively influence the oil quality and shelf-life, since it can lead to oil photooxidation due to the chlorophyll’s role as a sensitizer [64]. To obtain a qualitatively better oil, it is possible to use an alternative extraction process such as supercritical fluid extraction (SFE), ultrasound assisted extraction (UAE), or microwave assisted extraction (MAE). SFE is based on the use of a gaseous medium above or near its critical temperature and pressure to recover extracts from solid matrices. Carbon dioxide (CO_2_) is the most commonly supercritical fluid used in the food industry for extraction from plant materials. Aladìc and colleagues [64] demonstrated that this method of extraction can extract more efficiently the tocopherols than the traditional cold-pressing, leading to two- or three-fold higher γ-tocopherol content in the extracted oil. Another alternative extraction process that has been shown to improve both the yield and nutritional quality of the hempseed oil was the enzyme-assisted cold-pressing (EACPM) method. Indeed, Latif and co-workers [65] found that, by using this extraction technique, the level of total tocopherols in the hempseed oil increased from 4.8 to 14.1% (based on the enzyme’s type) in comparison to that obtained by the cold-pressing method. Finally, UAE and MAE resulted even more efficient in increasing the extraction not only of tocopherols (+17% and +11%, respectively) but also of other oil-soluble antioxidant compounds present in the hempseeds, such as polyphenols, since the oil obtained through these techniques were shown to have higher oxidation stability and antioxidant capacity than that extracted by SFE. This has been attributed either to the capacity of both UAE and MAE processes to extract oil at a lower extraction temperature and a shorter process time [60,61]. These methods can extract oil and oil-soluble components from seeds using the effect of ultrasounds and microwaves, respectively, on the seeds. The ultrasounds, propagating through the cells, generate the phenomenon of cavitation, which provides a greater solvent penetration into the sample matrix, increasing the contact between the sample and the solvent, and thereby, enhancing the efficiency (yield) and reducing the time of extraction, whereas, the microwaves can easily penetrate into the sample pores causing the solvent trapped in the pores to heat evenly and rapidly; in this way, the kinetics of extraction increases, leading to shorter extraction times, higher extraction rates, lower costs, and less solvent use.

The extraction technique can have an influence on the tocopherol amount of the oil but not on that of the whole hempseed. Factors that mainly affect the tocopherols amount in the seeds and indirectly, also in the derived oil, are both genotype and, above all, environmental factors. About this, Irakli and colleagues [3] observed that the differences found in the tocopherols amount of seven industrial hemp *cvs* grown in Greece in three consecutive years, were mainly due to the effect of growing year (71% of the total variance) than the genotype (24% of the total variance). This, adding to the use of different extraction techniques used for the tocopherol analysis from hempseed, could explain the much lower tocopherol amount found by the authors in comparison to the very high tocopherols concentration obtained by Vonapartis and co-workers [34].

Phytosterols are fat-soluble compounds found only in plants; they cannot be synthesized in human and are characterized to have a structure similar to that of cholesterol. Thanks to this chemical feature, when phytosterols are ingested, in the intestine, they are able to reduce the cholesterol solubility, excluding it from lipid micelles and to compete with free cholesterol uptake, thereby preventing the cholesterol intestinal absorption. To date, only three reports are investigated the phytosterols content and profile of whole hempseed [49,50] or hempseed oil [57] (Table 5).

As noted for tocopherol content, the total phytosterol content is more concentrated when expressed for hempseed oil rather than for whole seeds. Anyway, the most abundant phytosterol has been shown to be β-sitosterol with a value of 190.5 mg/100 g for hempseed oil [57] and 79.7 [50] and 53.61 mg/100 g [49] for the whole hempseed belonging to two different *cvs.* β-sitosterol in the seed plays an important role in the fluidity of plant cell and membrane and has a role in cellular differentiation. In humans, it can be effective to reduce hypercholesterolemia; can have favorable effects against colon cancer; and possesses antiviral, antifungal, and anti-inflammatory properties [50]. According to Vecka and colleagues [50], the amount of β-sitosterol in whole hempseed is higher than in linseed (79.7 vs. 55.4 mg/100 g seeds) and it is similar to the richest source of β-sitosterol analysed in the same study, namely pistachio (85.9 mg/100g seeds). The other two main phytosterols found in hempseed are campesterol and stigmasterol. The latter was described as a compound able to inhibit several pro-inflammatory factors and potentially effective in the prevention of osteoarthritis [50].

Finally, the other still little explored components of the unsaponifiable matter of hempseed oil are carotenoid. Analysis of total carotenoids has been performed only bya few papers [48,64,66] on the hempseed oil obtained by CO_2_ SFE and cold-pressing process, whereas data on the carotenoids profile on whole hempseed are reported only by Irakli and colleagues [3]. According to these papers, the total carotenoids of hemp oil has been found to range from 7.8 to 8.2 mg/100 g for oil obtained by two different cold-pressing methods applied on Hlesiia *cv* [48] and amount to 3.15 or 12.54 mg/100 g for oil obtained by cold-pressing and CO_2_ SFE, respectively [64]. In another work [66], the amount of total carotenoids in hemp oil obtained by CO_2_ SFE was 3.42 mg/100 g. These differences may be due to the adoption of different CO_2_ SFE parameters as well as genotype and environmental factors as reported in the publication of Irakli and colleagues [3]. As said, this is the only one report that analysed the carotenoid profile of whole hempseeds belonging to seven different *cvs*, showing that all analysed varieties contained the three main carotenoids lutein, zeaxanthin, and β-carotene, with lutein as the most abundant carotenoid ranging from 1.4 to 3.4 mg/100 g seeds, followed by β-carotene (0.2–0.8 mg/100 g seeds) and zeaxanthin (0.2–0.5 mg/100 g seeds). In this case, the total carotenoid content was found ranging from 1.4 to 4.3 mg/100 g seeds with values significantly affected by both genotype and growing year.

#### 3.1.2. Hempseed Proteins

The protein content of whole hempseed can vary from 20 to 25% (Table 2) according to variety and environmental factors. This amount can further increase in some hempseed-processed products such as dehulled seed and hempseed meal or cake (also called hempseed flour), that is, the remaining fraction of hempseed obtained after expelling its oil fraction [45,46,48,49]. Mattila and colleagues [45] demonstrated that in hempseed the proteins are mostly located in the inner layer of the seed, in fact they found only a low quantity of total proteins in the hull. Therefore, the increase in the protein content of processed products can be explained as a consequence of protein concentration after removing some component of the whole seed that totally or almost lacks in protein, such as the hull, where most of the fibre is located and the removal of which leads to a 1.5 times increase in both protein and oil amount. More proteins are also present when both hull and oil (the major component of whole hempseed) are removed. Indeed, their removal leads to obtaining the hempseed-processed product with the highest protein (up to over 50%) and the lowest fat content (even less than 10%, based on the type of extraction methods used) [46].

Research about the hempseed proteins originated from the early 20th century and highlighted that the two main proteins of hempseed are the storage protein albumin, a globular protein, and edestin, a legumin. This latter is the most abundant component, constituting about 82% of total hemp protein content. It is a hexamer of about 300 KDa composed by six identical subunits, each of which is in turn made up of an acidic (AS) and a basic (BS) subunit with molecular weights of about 33.0 and 20.0 KDa, respectively, that are linked by one disulphide bond [67,68]. In addition to edestin, the other components that have been found were albumin components (13% of total protein) and β-conglycinin (a vicilin-like protein) (up to 5% of total protein), the presence of which has been recently confirmed also by Pavlovic and co-workers [38] and in a recent genome-wide study [69] in which two albumin and one vicilin-like encoding genes in addition to three edestin isoforms where identify, each encoded by a different gene, differing for the amount of sulphurated amino acids that are particularly abundant in the isoform-type 3. Nevertheless, in the report of Pavlovic and co-workers [38], the authors found that edestin amounted to about 65% of total hempseed proteins. This result is in line with what was found from the HPLC and MS/MS analysis performed by Mamone and colleagues [70], but it is less than what was obtained in the previous studies by Tang and colleagues [67] and by Wang and co-workers [68]. Moreover, these latter authors also found that the solubility of hempseed proteins at acidic pH was lower than that of soy proteins, and this may be due to the formation of covalent disulphide bonds among individual molecules of edestin, resulting in insoluble protein aggregates. Another physio-chemical property investigated was the thermal one. It was found that the denaturation temperature of hempseed proteins was 92 °C. This evidence is in agreement with what was observed by Raikos and colleagues [71] about the effect of heat treatment (80 °C or over) on the structural characteristics of hempseed proteins and consequently, on their digestibility. Indeed, high temperatures can lead the proteins to unfold and to expose their hydrophobic groups, favouring protein-protein interactions instead of the protein-water ones, and, hence, the formation of insoluble protein aggregates to which digestive enzymes cannot access. In this context, Wang and co-workers [68], investigating on the in vitro digestibility of hempseed protein isolates, had previously shown that untreated hempseed proteins were much more digestible compared to soy proteins. The high digestibility of hempseed proteins has been confirmed also by Mamone and colleagues [70], who observed that only a handful of peptides survived to an in vitro digestion process. On the contrary, Lin and colleagues [72] observed that heat pre-treatment on hempseed proteins at 100 °C for 15 or 30 min, improved their digestibility. The authors explained this evidence as a consequence of the improvement in the digestive enzymes’ bioaccessibility on target proteins through increased exposure of susceptible peptide bonds after protein unfolding.

Regarding the nutritional value of hempseed proteins, it is important to consider that the nutritional quality of a protein is defined by its amino acid composition and by its digestibility and bioavailability. The protein’s amino acid composition along with the individual’s amino acid requirement, are important to establish the amino acid score, which is the relative contribution that the amino acids contained in the protein meet the individual’s amino acid requirement, whereas, the protein digestibility is closely related to the bioavailability of its amino acids, since it measures the degree to which the protein is digested and their components—the amino acids—are absorbed by the gastrointestinal tract and, hence, introduced into the human body. Several authors investigated the hempseed proteins’ amino acid composition [45,46,47,67,68,73]. Some of them analysed the protein extracted from whole hempseed while others examined commercial hempseed protein isolate, so the obtained results are expressed on either a whole seed basis (g of amino acids per 100 g of seeds), or total protein basis (g of amino acids per 100 g of protein). In Table 6, the literature data about the amino acid composition for whole hempseed are reported.

Amino acid composition obtained for hempseed proteins by various authors, are in good agreement and highlighted (1) that hempseed proteins contain all essential amino acids (EAAs) required by humans, and (2) that the most abundant amino acid is glutamic acid (3.74–4.58% of whole seed) followed by arginine (2.28–3.10% of whole seed). As previously anticipated, several authors [47,67,68] compared the amino acid composition of hempseed proteins to that of soy proteins and casein. The soy proteins have been chosen since they are one of the most important and commonly used plant sources of high-quality proteins [67], whereas casein is a highly digestible animal protein, considered a complete protein source, and therefore, it is commonly used as a reference protein to assess the nutritional quality of other proteins [46]. Moreover, soy proteins and casein are considered good sources of amino acids for infants. From this comparison, it emerged that hempseed proteins have a good amount of sulphur-containing amino acids, higher than both casein and soy proteins. Furthermore, in comparison to soy proteins, the amino acid content of hempseed proteins was higher or similar, except for aspartic acid, glutamic acid, and lysine that resulted in being higher in soy proteins, whereas, if compared to casein, the amino acid content of hempseed proteins was higher or similar, except for tyrosine, leucine, methionine, and lysine that were more abundant in casein. Among EAAs, only three (isoleucine, lysine, and phenylalanine) resulted in being lower in hempseed proteins in comparison to casein, while the others appeared to be higher or similar and the proportion of EAAs to the total amino acids for hempseed proteins resulted in being higher than soy proteins and similar to casein. However, to understand if a protein can satisfy the human nutritional requirement, it is necessary to estimate the amino acid score for each amino acid element. The amino acid score for hempseed proteins has been calculated in different papers [46,67,68] based on the Food and Agriculture Organization/World Health Organization (FAO/WHO) amino acid suggested requirements for children 2–5 years old. The results obtained from all reports agree with the evidence that lysine was one of the limiting amino acids since its score was lower than 1 (0.75 in [67]; 0.72 in [68]; and 0.62 in [46]). Instead, conflicting results were found for other limiting amino acids of hempseed proteins. In both the papers of Tang and Wang and colleagues [67,68], others limiting amino acids were shown to be the sulphur-containing amino acid (methionine + cysteine) with an amino acid score of 0.65 [67] and 0.62 [68]. Hence, comparing the sulphurated amino acid score with the lysine amino acid score, the former resulted in the first limiting amino acid followed by lysine. Contrarily, House and co-workers [46] found that lysine was the first limiting amino acid followed by tryptophan (amino acid score, 0.87) and leucine (amino acid score, 0.94) and the limitation in the lysine content of hempseed proteins, ranks hempseed in the same range of the main cereal grains. These differences in the results may be related to the fact that the analyses were performed on different type of samples (i.e., commercial hempseed protein isolates and proteins extracted from whole hempseed). Tang and colleagues [67] have also calculated the amino acid score based on the FAO/WHO amino acid suggested requirements for children 10–12 years old and for adults, showing that, in these cases, there are no limiting amino acids.

House and colleagues [46] investigated the in vivo digestibility of hempseed proteins using a rat bioassay for protein digestibility, with the aim to evaluate the actual amino acid bioavailability. The authors observed that the in vivo protein digestibility of hempseed proteins collocates hempseed in the same range as the major pulse protein sources, such as lentils, and above cereal grain products. Furthermore, they also found that the presence of hull in the seed negatively affects the digestibility of the hempseed proteins. In fact, removal of the hull fraction from the hempseed led to an average increase in the protein digestibility from 85.2 to 94.9%. The authors supposed that the high fibre content of the hull may exert an influence on the digestibility of the proteins, but it is also possible that this decrease is, almost in part, due to the presence of some antinutritional factors such as phytate and trypsin inhibitors [39,74,75], with a different distribution in the various parts of the whole hempseed (see Section 3.1.5).

Overall, whole hempseed can be considered a rich-protein source containing a protein amount higher or similar than other protein-rich products, such as quinoa (13.0%) [45], chia seeds (18.2–19.7%) [76], buckwheat seeds (27.8%) [45] and linseeds (20.9%) [45]. Nutritionally, the protein fraction of hempseed is highly digestible, has a good profile of EAAs required for infants similar to those of casein except for lysine that is the first limiting amino acid in hempseed proteins, even if this limitation is related only for the amino acid requirement for infants up to 5 years old who require a larger proportion of lysin. Furthermore, in addition to EAA profile, it is important to consider also the benefits provided by the amount of other non-EEAs such as arginine. In fact, arginine is a dietary precursor for the formation of nitric oxide (NO), a potent mediator of vascular tone, and therefore it is very important for the health of the cardiovascular system. Additionally, arginine and NO specifically, have been linked to optimal immune function and to muscle repair [46], so from this point of view, the proteins of hempseed represent an important source of digestible arginine. Hence, whole hempseed or their derived products such as hempseed meal or hempseed protein isolate can be considered a good viable, vegetable-based protein source for human diet.

#### 3.1.3. Hempseed Carbohydrate and Dietary Fibre

Dietary fibre is defined as the part of plant material in the diet, which is resistant to enzymatic digestion and that includes cellulose, non-cellulosic polysaccharides such as hemicellulose, pectin, gums, mucilage and a non-carbohydrate component, namely lignin. Nutritionally, the dietary fibre is considered an integral part of the carbohydrate fraction of a food matrix. As previously said, the total carbohydrate content of hempseed can range between 20 and 30% (Table 2). Actually, only a few literature reports analysed the total carbohydrate and fibre of hempseed. Among these, Callaway [47] found that the total carbohydrate content of the whole hempseed belonging to the Finola *cv* amounted to 27.6 g/100 g of seeds, whereas Mattila and colleagues [45] by analysing the nutritional value of some commercial protein-rich seeds, among which is hempseed, found that the total carbohydrate content of whole hempseed was similar to those found in the whole flaxseed (34.4 ± 1.5 g/100 g of seeds and 29.2 ± 2.5 g/100 g of seeds, respectively). However, the most interesting result obtained in both studies was that a great part of the total carbohydrate of the analysed hempseed was constituted by dietary fibre, the most of which was the insoluble fraction (Table 2). In particular, Callaway found a Total Dietary Fibre content (TDF) of 27.6 g/100 g of seeds [47], demonstrating that the entire carbohydrate fraction consisted in dietary fibre, whereas in the study of Mattila and co-workers [45], the TDF of hempseed amounted to 33.8 ± 1.9 g/100 g of seeds, representing the 98% of the total carbohydrate. Since the remaining carbohydrate after excluding the amount of TDF is essentially starch, it is possible to assert that hempseed represents a low-starch content food matrix and a good source of dietary fibre, above all, the insoluble fraction. The high content of Insoluble Dietary Fibre (IDF) was found also by Multari and co-workers [77] for hempseed flour, which resulted in an IDF content of 25.49 g/100 g, whereas the Soluble Dietary Fibre (SDF) content was 0.16 g/100 g, thus highlighting that hempseed is one of the richest sources of IDF among several high-protein crops such as green pea, buckwheat, and fava bean (IDS, 8.69 ± 0.07 g/100 g, 6.98 ± 0.01 g/100 g, and 9.39 ± 0.30 g/100 g, respectively). These results were rather consistent with those obtained by Callaway (IDF, 22.2 g/100 g; SDF, 5.4 g/100 g) [47] and by Mattila and colleagues (IDF, 30.9 ± 1.5 g/100g; SDF, 2.9 ± 0.4) [45] for the amount of SDF and IDF in whole hempseed.

An estimate of the IDF could be obtained also through the analysis of Neutral Detergent Fibre (NDF), which measures the amount of the cell wall components, namely, hemicellulose, cellulose and lignin. Vonapartis and colleagues [34] reported that the average concentration of NDF in hempseed of ten industrial hemp *cvs* grown in Canada, was 35.7 g/100 g and this result was in good agreement to that obtained by House and colleagues (NDF, 32.1 g/100 g) [46]. To note, it has been demonstrated that much of the fibre fraction of whole hempseed is located in the hull, as the fibre content obtained for dehulled hempseed was significantly lower than that found for the whole hempseed [45,46].

As previously said, the high fibre content of whole hempseed can negatively affect the protein digestibility; however, it is also important to take into account that the consumption of dietary fibre provides several health benefits in the human body. Indeed, dietary fibre, including the insoluble fraction, is considered a functional product acting as a probiotic, among others. In particular, it has been shown that it can improve insulin sensitivity; can reduce appetite and food intake, thus decreasing the risk of obesity and diabetes; and can lower the blood total cholesterol and low-density lipoprotein (LDL); moreover, because of the dietary fibre resists to digestion in the small intestine, it reaches the large intestine, where it is fermented by the gut microbiota, to produce short chain fatty acids with anti-carcinogenic and anti-inflammatory properties [78].

Hence, considering the health benefits derived by the ingestion of dietary fibre together with the considerable amount of fibre in hempseed, it would merit consideration as an ingredient to enrich the fibre content of foods, thereby improving their nutritional value. In this context, it should be taken into account that the use of whole hempseed (as it or defatted) would be more appropriate since almost all the fibre is located in the hempseed hull.

#### 3.1.4. Hempseed Minerals

The total mineral content of a food matrix is indicated by the amount of ash that is the inorganic matter of the sample. Minerals are considered as micronutrients because their dietary requirement is relatively low (1–2500 mg/day depending on the mineral’s type); nevertheless, they are needed to maintain optimal health, playing physiological and structural essential roles. Based on their dietary requirements, minerals can be classified as macro-elements (i.e., minerals needed in amount of >50 mg/day), including phosphorous, potassium, magnesium, calcium; and sodium, and as micro-elements or in-trace elements (i.e., minerals needed in amounts of <50 mg/day) like iron, manganese, copper, and zinc.

Although different authors analysed the total ash amount in several varieties of hempseeds (Table 2), highlighting that they are a good source of total minerals also considering other oilseeds like chia seeds [76] and linseeds [79] (4.9–6/100 g for hempseeds, 4.56–5.07 g/100 g for chia seeds and 3.5 g/100 g for linseeds), only a few authors investigated their minerals profile [43,44,45,47,48,49] showing that they can be considered a good source of useful macro- and micro-elements. The results obtained by these authors are reported in Table 7.

In general, the mineral profile of seeds can widely vary based on environmental condition, mineral soil composition, the use or not of fertilizers, the type of fertilizer if used, as well as the plant variety. However, except to Siano and colleagues [49], other authors who have investigated the mineral profile of hempseeds belonging to different varieties and grown in various countries, obtained consistent and comparable values [43,44,47,48]. The major macro-elements found in hempseeds were phosphorous (P), potassium (K), magnesium (Mg), calcium (Ca), and sodium (Na), whereas among the in-trace elements, iron (Fe), manganese (Mn), zinc (Zn), and copper (Cu) have been reported.

Considering either EFSA’s Dietary Reference Value (DRV) [80] and the Food and Nutrition Board of the Institute of Medicine’s Dietary Reference Intake (DRI) [44], and the mineral content of hempseeds reported for different hemp *cvs*, it is possible to claim that hempseeds represent an excellent natural source of P, K, Mg, Ca, Fe, Zn, Cu, and Mn. Particularly, the amount of P, the most abundant mineral found in hempseeds, resulted in being higher also than that found in niger seeds (*Guizotia abyssinica* (L.f.) Cass.) and linseeds (*Linum usitatissimum* L.), which are oilseeds like hempseeds and are considered optimal phosphorous’ sources (P average content, 784.64 mg/100 g and 461.35 mg/100 g, respectively) [81]. The level of K in hempseeds is in general higher compared to that found in linseeds (568.91 mg/100 g) [81], and is equivalent to that observed in hazelnut (863 mg/100 g), thought to be an optimal source of this macro-element [82]. Interestingly, the high K amount along with a relatively low Na content, leads to a high K/Na ratio, which is believed to be related to cardioprotective effects as it promotes a high K intake considered to be inversely related to blood platelet aggregation and stroke incidence. Mg also contributes to hearth function and health, so that a Mg deficiency can lead to cardiac dysfunction. The amount of this mineral in hempseeds results in being similar to walnut (Mg range: 381–443 mg/100 g) which is one of the most important sources of Mg [83]. Among the in-trace elements, Fe is of particular importance considering its essential role for human health and its widespread dietary deficiency. Interestingly, the hempseed’s Fe content resulted to be exceptionally high in the study of Mihoc and colleagues [43] in comparison to others works (Table 7), but this may be due to the very high Fe level in the experimental growth soil (1900–2043 mg/100 g). Lan and co-workers [44] highlighted that the hempseed’s Fe content is much higher than cereal grains, and for this, hempseeds could be used to enrich cereal food products, ameliorating iron deficiency. In the same study, the authors have also calculated the percent contribution of minerals per serving of hempseeds (30 g of seeds) to Reference Daily Intake (RDI) for adult males from age 19 to 30, finding that the highest % RDI supplied by hempseeds of the analysed industrial hemp *cvs*, was for Fe and Mn (average % RDI, 46.68 and 169.14, respectively), concluding that all analysed varieties represent an excellent sources especially of Fe, Mn, Cu, Zn, P, and Mg. Additionally, it has been shown an increase in the content of some minerals in hempseed kernel after dehulling. Particularly, in the Hungarian Hlesiia *cv*, hull removal led to an increase in phosphorous (1.5 times), iron (1.25 times), and zinc (2 times) [48]. Whereas Mattila and co-workers [45] observed that whole hempseed held 30–65% fewer macro-elements and Zn among in-trace elements in comparison to hemp hulls, whilst Cu and Mg were more evenly distributed in both the seed’s kernel and hull.

It is known that hemp plant with its wide root system is among the plants appropriate for phyto-depuration, namely decontamination of soil from the high and toxic level of heavy metals such as cadmium (Cd). For the non-smoking population, the major source of exposure to Cd is food and among the most important Cd food sources can be mentioned integral cereals, potatoes, seafood, fungi, and oilseeds. A dietary exposure to Cd in humans leads to a relatively low, but efficient absorption of the metal in the kidney and liver, where it has a very long biological half-life ranging from 10 to 30 years. Cd is primarily toxic to the kidney, especially to the proximal tubular cells, where it accumulates over time and may cause renal dysfunction. Cd can also cause bone demineralization, either through direct bone damage or indirectly, as a result of renal dysfunction. Moreover, the International Agency for Research on Cancer has classified Cd as a human carcinogen (group 1) after that data on human exposure to Cd in the general population have been statistically associated with increased risk of some cancer types such as lung, endometrium, bladder, and breast cancers [84]. Taking into account the high capacity of hemp to absorb minerals from soil, including heavy metals, Mihoc and colleagues [43] evaluated the hemp plant capacity to translocate the absorbed Cd into the seeds in a soil with high Cd content (>0.3 mg/100 g) in the absence or presence of one of two different fertilizers (K and P fertilizers). They analysed the seeds belonging to five industrial hemp *cvs* approved for growth in Romania and they showed that hemp is able to absorb and translocate to the seeds from 130 to 400 µg/100 g of Cd, with the highest values obtained for plants treated with both K and P fertilizers. Considering the highly cadmium toxicity for human health, the European Food Safety Authority’s Panel on contaminants in the food chain [85] has set the tolerable weekly intake (TWI) for cadmium at 2.5 µg/Kg of body weight, whereas the FAO/WHO recommended 100 µg/Kg in cereals and legumes and up to 300 µg/Kg in medicine as the cadmium upper limit for safe human consumption [43]. Hence, the value of Cd found in hempseeds by Mihoc and colleagues [43] far exceeds the upper limit set by the FAO/WHO, even if according to the EFSA TWI value, for an adult man with an average body weight of 70 Kg, the TWI for Cd is 175 µg, and 30 g (a serving portion) of hempseeds belonging to plants grown in a soil with high Cd content, has a Cd level below the TWI (39–120 µg of Cd). In addition, the medium Cd content in soil is generally of 0.05–0.08 mg/100 g, namely from 6 to 37.5 times lower than that used in the work by Mihoc and colleagues [43]. Indeed, in another work investigating the heavy metal content in twenty-one hempseed samples collected from north western Turkey, the Cd content in the samples was found to be within the range of 0.5–2.3 µg/100 g, much lower than the upper limit recommended from the FAO/WHO [84]. Similarly, Mattila and colleagues [45] observed that the Cd level in whole hempseed was 1.5 µg/100 g. In their study, Mihoc and co-workers [43] also investigated whether and how the minerals’ soil composition, the presence or absence of different fertilizers and the seeding distance could influence the minerals profile of the analysed hempseeds. They observed that both fertilizers at different levels of administration, decreased the absorption of Zn and Mn due to both the soil’s pH increase which limited the availability of minerals from soil, and the mineral-phosphate complexes forming for the P excess owing to the use of fertilizer. Furthermore, the authors showed that the content of Ca, Mg, and K in the seeds was mainly affected by varieties. The uptake of Ca by the plants was disturbed also by fertilizer, whereas K uptake was influenced also by further factors, such as seeding distance and environmental factors such as soil moisture and temperature.

#### 3.1.5. Hempseed Antinutritional Compounds

The antinutritional factors are biological compounds present in human or animal foods that reduce nutrients’ bioavailability or food intake, or the metabolism of which may lead to the release of toxic products, thereby contributing to impaired gastrointestinal and metabolic performance. Generally, compounds such as saponins, phytic acid, alkaloids, certain oligosaccharides, protease inhibitors, cyanogenic glycosides, glucosinolates, and tannins are traditionally incorporated into this group. Referring to hempseed, only a few reports [39,73,74,75] investigated about the antinutritional compounds in this seed, and the antinutritional factors that have been considered were phytic acid, trypsin inhibitors, condensed tannins, cyanogenic glycosides, and saponins. The literature data about these analyses are shown in Table 8.

Phytic acid is a natural plant compound acting as the major storage form of P as well as the primary P reserve for the plant. Indeed, it constitutes 60 to 70% of the total P in cereal grains, oilseeds, and legumes and generally comprises 1–5% weight in cereals, oilseeds, legumes, and nuts [86]. From a chemical point of view, PA is a myo-inositol hexakisphosphate (IP6) composed by an inositol ring esterified with six phosphate groups that are not internally connected; hence it has up to 12 replaceable protons allowing it to bind with multivalent cations (i.e., Zn^2+^, Fe^2+/3+^, Ca^2+^, Mg^2+^, Mn^2+^, and Cu^2+^), positively charged proteins, and in some cases, starch through hydrogen bonds [87], forming insoluble complexes known as phytates. Both phytate-mineral and phytate-protein complexes are not readily absorbed by monogastric animals including humans because of their inability to metabolize phytic acid due to the lack of a sufficient level of phytate-degrading enzymes in their digestive tract. Thus, these complexes negatively affect the bioavailability of dietary and endogenous minerals and proteins as well as the digestive enzymes’ activity; therefore, for these reasons, phytic acid is considered an antinutrient. However, phytic acid may also have beneficial effects in the prevention and treatment of several pathological conditions related to oxidation and cancer [74], maybe due to its ability to chelate Fe ions, that lead phytic acid to inhibit iron-driven hydroxyl radical (–OH^•^) formation and, hence, to strongly suppress lipid peroxidation and the oxidation of other biomolecules. Hence, for optimum physiological benefit of phytic acid, the phytate content in the upper part of the gut should be low to avoid negative effects on mineral and protein absorption and bioavailability, but at the same time, it should be high enough to use its antioxidant and anticancer activity. However, to date, accurate and clear information on the phytate dosage required to elicit beneficial effects in human is still limited. In accordance with Russo and Reggiani [73], to increase the safety for feed and food formulation, it is preferable to reduce the phytic acid content to at least 0.45 g/100 g of defatted sample. According to literature data, the results obtained by different authors about the phytic acid content in hempseed (Table 8) are in good agreement and show that the level of this antinutritional compound ranges between 4 and 8% of the dry weight of the defatted matter. Galasso and colleagues [39] found a significant variability in the phytate content among different hemp *cvs*, and Russo [75] showed that French monoecious varieties had lower content than Italian dioecious ones, but this evidence is exactly the opposite of what Galasso and co-workers [39] found analysing twenty hempseed *cvs* and accessions of different origin, among which, the French varieties have been to be those with the highest phytic acid content. These differences in the results could be explained by the significant influence of genotype, growing year and the interaction between these two factors on the antinutritional compounds’ amount, phytic acid included [73]. Compared to other seeds, the content of phytic acid in hempseed seems to be higher. For example, Russo and Reggiani, and Russo and co-workers [73,75] highlighted that the phytic acid content of hempseed was higher than those found in soy bean (2%), and Mattila and colleagues [74], comparing different type of oilseed, legumes, and pseudo-cereal grains, observed that oilseeds in general, including hempseed, linseed, and rapeseed, form a group with a higher phytic acid content in comparison to other legumes (e.g., fava bean and lupin) and pseudo-cereal grains (e.g., quinoa and buckwheat), and among the analysed oilseeds, whole hempseed was that with the highest phytic acid level.

Condensed tannins (CTs) are phenolic compounds (flavonoid oligomers, specifically), ubiquitous in the plant kingdom, designated as proanthocyanidins. They are considered as antinutritional compounds because of their ability to form insoluble complexes with proteins and minerals, negatively affecting nitrogenous compounds uptake and absorption of minerals [73], and thus reducing the nutritional value of foods. Moreover, based on their concentration, CTs can affect the palatability of the meal. Nevertheless, being phenolic compounds, in the monomeric form, they also have some beneficial effects on health acting not only as antioxidants, but also as anticarcinogenic, cardioprotective, anti-microbial and anti-diabetic compounds [74]. According to Russo [75], the attention threshold for CTs is 1% of dry weight of the product. Although the amount of CTs found in hempseed meal (Table 8) has been shown to be higher than other plant sources, for example soy (10 mg/100 g), the hempseed meal’s CTs content is however below than the attention threshold, so it neither is toxic, nor affects the hempseed meal palatability [75]. Russo and Reggiani [73] found significant variability for the CTs content in monoecious and dioecious *cvs*, with a double level in the monoecious varieties than dioecious ones. Mattila and co-workers [74] evaluating the amount of CTs in whole hempseed and in the hull of hempseed, observed that the hull had slightly higher CTs content than whole hempseed, even if the difference was rather small (105 ± 4 mg/100 g and 144 ± 5 mg/100 g, respectively). The same authors also found that CTs in hempseed were essentially epi-catechin polymers (i.e., procyanidins).

Trypsin inhibitors (TIs) are a type of endogenous antinutritional factor commonly present in various crops, seeds, and vegetables, among which are pulses and legume seeds, nuts, oilseeds, and cereal grains. They can have a negative impact on the nutritional value of food and feed products as they affect protein utilization and digestion in human and animals. Their role in the plant is to protect the plant against microbial proteinases and to regulate the activity of endogenous proteinases. From a nutritional point of view, the ingestion of TIs reduces the digestibility of food proteins and the bioavailability of amino acids because they can pass unaltered through the stomach as they are stable against pepsin and at low pH [88], and in the small intestine, they are able to inhibit the biological activity of the digestive enzymes trypsin and chymotrypsin—both important for the digestion of protein in living organisms—by competitive binding and in this way, they lead to the formation of an irreversible trypsin/chymotrypsin enzyme-TI complex. TIs have been largely studied mainly in legume seeds, particularly in soybean, as it is one of the richest sources of these antinutrients. Guillamòn and colleagues [89], investigating the TIs content in different types and varieties of legume seeds, identified three groups of legume seeds according to the TIs content:

- the highest content group including different soy (*Glycine max*) *cvs* with a TIs content ranging from 43 to 84 U/mg of the sample;
- the medium content group corresponding to common bean (*Phaseolus vulgaris*) varieties containing from 17 to 51 U/mg of the sample; and
- the lowest content group including lentil (*Lens culinaris*) and fava bean (*Phaseolus vulgaris*) *cvs* containing from 3 to 10 U/mg of the sample.

Although most of the works regarding TIs are performed on soy and legume seeds in general, some literature data about the amount of these antinutritional compounds in oilseeds such as Sacha inchi (*Plukenetia volubilis* L.) [90] and linseed [91] are also reported. The TIs amount in hempseed meal reported by different authors (Table 8) was found to range between 10.8 and 28.4 U/mg, and the differences in the TIs content is probably due to both genotype and growth conditions [39,74,75]. These values are similar to those found for the TIs content obtained for linseed meal (13.8–24.7 U/mg) by Russo and Reggiani [91] and, considering the grouping made by Guillamòn, they would place both linseed and hempseed in the medium content group of TIs. Interestingly, Pojić and co-workers [92] highlighted that the inner fraction of hempseed, containing predominantly cotyledon particles, is the part of the seed with the highest TI activity compared to the fraction containing the hull particles. By contrast, Mattila and colleagues [74] found that the TI activity between whole hempseed and hempseed hull was similar, indicating that TIs are equally located through the hempseed’s kernel and hull.

Cyanogenic glycosides are nitrogen-containing secondary metabolites that serve to the plant as a protective device against predators such as the herbivores. The level of cyanogenic glycosides produced by the plant is dependent upon the age and variety of the plant, as well as the environmental factors [93]. They are considered toxicants for humans because, structurally, they are glycosides of 2-hydroxynitriles that can be hydrolysed by the enzyme β-glucosidase into cyanohydrin, which is unstable and thus dissociates, leading to the toxic hydrocyanic acid. Although this is a violent poison, oral intake of cyanogenic glycosides (e.g., via food) is not necessarily toxic, particularly in the short-term intake. Indeed, hydrolysis of the cyanogenic glycosides in the digestive tract or by the liver leads to a slow release of hydrocyanic acid that is readily detoxified by the body. According to Russo [75], at levels below 100 ppm, the cyanogenic glycosides are not harmful to health. The amount of these antinutrients in hempseed has been investigated only in two reports [73,75], showing that, in the hempseed meal, their level significantly varies according to genotypes [75] and monoecious or dioecious *cvs*, with monoecious varieties containing lower cyanogenic glycosides than dioecious ones [73]. Moreover, these studies highlighted that the hempseed meal of some hemp *cvs* has a cyanogenic glycosides content above the safety limit, but since this value refers to hempseed meal, it is resulted to be more concentrated compared to the whole seed.

Finally, saponins comprise a large family of structurally related compounds containing a steroid or triterpenoid aglycone (sapogenin) linked to one or more water-soluble oligosaccharide moieties. At larger quantities, these compounds are gastric irritants, and they can exert toxic properties causing haemolysis of red blood cells. However, at low doses, they may have a plasma cholesterol lowering effect in humans, as well as strong cytotoxic effects against cancer cell lines, and are important in reducing the risk of many chronic diseases [74]. The FAO/WHO established a safety limit of 12% of saponins for quinoa seeds which are among the main saponins’ plant sources [94]. Russo and Reggiani [73,75] found that the saponins amount in hempseed meal of different *cvs* was well below than 12%, and lower than other plant sources such as linseed and soy, despite the results they obtained from two separate studies being very different, with the level of saponins showed in one work [73] almost ten-fold higher than that obtained in the other work [75].

Concluding, among the various antinutritional compounds analysed in hempseed, TIs and, above all, phytic acid were the most abundant, based on a comparison with other edible seeds. Overall, it has been highlighted that all the analysed antinutritional compounds are positively correlated, and therefore, it is possible that the biosynthetic pathways of these compounds in hemp are expressed simultaneously during the seed’s development [73].

### 3.2. Hempseed Functional Features

The functional properties of hempseeds are related not only to their high nutritional value which awards them important beneficial features for human health, but also to the presence of different bioactive compounds, among which are unique phenolic compounds with antioxidant, anti-inflammatory, and neuroprotective actions, as well as bioactive peptides that will be discussed in detail in the next sections.

#### 3.2.1. Hempseed Phenolic Compounds

##### Composition Studies

Phenolic compounds are plant-derived secondary metabolites that are commonly produced by the plants as defence agents against biotic and abiotic stress, such as the attack of predatory species and UV light, respectively. These compounds, thanks to their chemical structures, have intrinsic antioxidant activity; therefore, they may protect cell constituents against oxidative damage and, hence, limit the risk of various degenerative diseases associated with oxidative stress. Indeed, in the human body they can exert a multitude of physiological activities, among which are cardioprotective and anti-inflammatory effects. Often, literature values about total antioxidant capacity and total phenolic content of a matrix are difficult to absolutely compare, due to the variety of analytical and extraction methods and the wide use of different reference compounds in the quantitation. Hence, in this review, we quantitatively compared the literature data obtained about these parameters by different authors, only if possible.

The first investigations about the antioxidant power and the total phenolic content (TPC) of hempseed were performed on hempseed oil. As previously said, the most abundant antioxidants in this product are tocopherols, especially the γ-isoform which confers to this oil a high oxidative stability despite the high level of unsaturated fatty acids that are highly susceptible to oxidation. However, together with tocopherols, also phenolic compounds contribute to the high oxidative stability of hempseed oil [52,58,95,96]. Indeed, it has been shown that hempseed cold-pressed oil represents a good dietary source of antioxidants which could have potential application in health promotion and disease prevention from oxidative damage, and therefore, it could be used to improve the quality and stability of food products. Smeriglio and colleagues [95] evaluating in detail the polyphenolic profile and the antioxidant properties of cold-pressed hempseed oil of the Finola *cv*, found that this product contained significant amounts of polyphenols, especially flavonoids such as flavanones, flavonols, flavanols, and isoflavones, the most representative and abundant of which were naringenin, kaepferol-3-*O*-glucoside, epicatechin, and daidzein, respectively. The presence of flavonoids in hempseed cold-press oil was demonstrated also by Faugno and co-workers [97] who investigated the polyphenol profile of cold-pressed oil of Uso 31 *cv*. The authors used the LS-ESI-MS/MS technique to assess the polyphenol qualitative and quantitative compositions of the oil and to investigate the influence of some agronomic practices on the quantitative variability of polyphenolic compounds. They found that, among phenols, the most representative compounds were dihydroxybenzoic acid, caffeoyl-tartaric acid isomers, *p*-coumaric acid, *N*-caffeoyltyramine, and *N*-feruoyltyramine, whereas, among the flavonoid subfamily, they found the glycosides of quercetin, kaempferol and isorhamnetin. Moreover, the authors observed that the highest production of polyphenols has been obtained without pre-seeding fertilization and with 60-plant/m^2^ crop density, whereas when the plant density was halved, the abundance of phenolic compounds was compromised. In general, as said, the quantitative variability of polyphenols is affected by the influence of multiple biotic and abiotic factors, which take place in the biosynthetic pathway of these compounds, as reported also by Irakli and colleagues [3], who highlighted that the amount of phenolic compounds in the whole hempseed is affected by genotype, growing year, and the interaction between these factors. However, the growing year and so, the environmental and agronomical conditions, were the factor that most affected the variability of the TPC (contribution to total variance, 59%). In Table 9, the literature data about the TPC extracted by different methods but analysed by the same technique (Folin–Ciocolteau) and expressed using the same reference compound (gallic acid) are reported, and they show the high variability of the parameters.

Although tocopherol was reported as the main antioxidant for hempseed oil, it would not be the same for the whole hempseed. Indeed, Irakli and co-workers [3] observed a significant positive correlation between the TPC and antioxidant activity of hempseeds belonging to seven industrial hemp *cvs* approved for cultivation in Greece and only weak correlation between γ-tocopherol and antioxidant activity, concluding that the antioxidant capacity of hempseed is due, mainly, to phenolic compounds rather than to the tocopherols. The high positive relation between the TPC and the radical scavenging activity of hydroalcoholic hempseed extract was also reported by Chen and colleagues [100]. These results can be explained by the fact that polyphenols are polar, hydro-soluble compounds and, thus, that only a few of them are isolated during oil extraction whereas most remain associated to the hemp cake [98]. Conversely, the tocopherols, that are fat-soluble, are extracted more efficiently, and hence, are more abundant in oil. This is in accordance with the results of Siano and co-workers [49] and Moccia and colleagues [98] who have analysed and compared the TPC and the radical scavenging activity of whole hempseed, hempseed flour, and hempseed oil of Fedora *cv*, showing that, although the values obtained for whole hempseed and hempseed flour were similar for both evaluated parameters, those for hempseed oil were significantly lower. Regarding the TPC of whole hempseed, Galasso and colleagues [39] highlighted that the value obtained for hempseed were slightly higher than those found for linseed (5.88–10.63 mg Caffeic Acid Equivalent (CAE)/g of dry weight of defatted hempseed vs. 4.64–9.40 mg CAE/g of dry weight of defatted linseed), which is considered a matrix rich in total phenols among the oilseeds [101]. In addition, the TPC and the total antioxidant capacity of whole hempseed could increase consequently the germination of seed, leading to a derived product–the hempseed sprouts—enriched with beneficial bioactive compounds [99,102].

Various authors found that in whole hempseed, polyphenols are mainly located in the hull rather than in the kernel [74,92,100] and that the main phenolic compounds that have been detected in *C. sativa* L. seeds are lignans, phenols derived from the shikimic acid biosynthetic pathway. Particularly, they originate from cinnamic acid derivatives which are biochemically related to the metabolism of phenylalanine. Lignans detected in hempseed are also called phenylpropionamides due to their chemical components and belong to two main groups known as phenolic amides and lignanamides [3]. The first ones are made by an ammine moiety (tyramine, derived from the tyrosine oxidative decarboxylation) linked to a phenolic moiety (a hydroxycinnamic acid, such as coumaric, caffeic or ferulic acid). The second ones are derived by a random polymerization of phenolic amides. Some of these phenols are found exclusively in the *C. sativa* L. plant. More than 20 kinds of phenylpropionamides, most of which are lignanamides, have been isolated from hempseed. Chen and co-workers [100] isolated and identified two hempseed lignans with strong in vitro antiradical activity and protective effect against in vitro oxidation of human LDL in an ethanol extract of hempseed hull. These compounds were the phenolic amide *N-trans*-caffeolyltyramine and the lignanammide cannabisin B, which were identified as the most abundant phenolic compounds in the hull fraction of hempseed also by Pojić and colleagues [92] who in addition, showed that the most abundant polyphenols in the cotyledon inner fraction of hempseed were catechin and *p*-hydroxybenzoic acid. The other phenylpropionamides identified as the most abundant in hempseed of different varieties were the lignanamides cannabisin A [3], cannabisin F, and grossamide [74]. Also, Yan and co-workers [103] isolated and identified a total of 14 phenolic compounds characteristic of the hempseed, twelve of which were lignanamides. Further phenylpropionamides recently isolated from hempseed were cannabisin I [104], cannabisin Q, and coumaroylaminobutanol glucopyranoside (CLG) [105], corresponding to the glycoside of 4-((E)-p-coumaroylamino)butan-1-ol previously described by Yan and colleagues [103].

##### Biological Effects

Concerning the functional effect, most of the phenolic compounds isolated from hempseed have been shown to have high radical scavenging activity compared to quercetin, and, in addition, the phenolic amide *N-trans*-feryoryltyramine and especially, the lignanamides 3,3′-demethyl-grossamide and 3,3′-demethylheliotropamide were also able to in vitro inhibit the acetylcholinesterase (AChE) enzyme at a concentration of 100 µg/mL, thus exhibiting properties similar to the medicines used for the treatment of mild to moderate Alzheimer’s disease (AD) such as galanthamine [103]. Bourjot and co-workers [104] found that, among the phenolic amides they have extracted from hempseed flour, *N-trans*-caffeoyltyramine had the highest antioxidant and arginase inhibitor activities. In this regard, the inhibition of arginase could raise NO bioavailability that ameliorates endothelial functionality and it can also reduce oxidative stress which plays a central role in the onset and progression of endothelial dysfunction involved in various diseases, including cardiovascular ones. However, the most important biological effects attributable to hempseed phenylpropionamides are the anti-inflammatory and neuroprotective activities. In this context, Zhu and co-workers [106] had recently isolated and characterized the chemical and functional features of two unique hempseed bioactive compounds named sativamides A and B. According to the authors, from a chemical point of view, they are non-lignanamide compounds characterized by a benzo-angular triquinane core (i.e., a core consisting of fused five-membered hydrocarbon rings and arranged in an angular position), derived from *N-trans*-caffeoyltyramine. Interestingly, it has been shown that pre-treating of the SH-SY5Y human neuroblastoma cell line with 50 µM of sativamides A or B, reduced the cell death caused by endoplasmic reticulum stress that has been shown to play an important role in neurodegenerative diseases, such as Parkinson’s disease and AD. The same cell line was also used by Maiolo and colleagues [107] to create a cellular model of Parkinson’s disease, on which the neuroprotective effect of various natural compounds, among which is *N-trans*-caffeoyltyramine extracted from hempseed, was tested. The obtained results highlighted that 150 µM of the hempseed’s phenolic amide was able to prevent the cell death induced by up to 150 µM of H_2_O_2_ treatment and due to oxidative stress and mitochondrial dysfunction caused by this toxic compound. Oxidative stress and mitochondrial dysfunction are thought to contribute to cell death in Parkinson’s disease; therefore, the resulting effect of 150 µM *N-trans*-caffeoyltyramine can be considered as potentially neuroprotective.

The neuroprotective effect of different hempseed phytochemicals has been shown to be related particularly to the anti-inflammatory and antioxidant action of some of these compounds on microglia cells which are immune cells of the central nervous system that are involved in regulating the immune responses in the brain, thus playing an important role in brain infection and inflammation. The persistent and/or over-activation of these cells is often related to neuronal damage and onset of neurodegenerative diseases, such as AD and Parkinson’s disease, and multiple sclerosis, that in fact, are characterized by chronic inflammation and oxidative stress. About this, Zhou and colleagues [105] investigated the anti-neuroinflammatory and neuroprotective action of each of the twenty phenolic compounds that they have isolated from hempseed, on the release of the inflammatory cytokine TNF-α from Lipopolysaccharide (LPS)-stimulated BV2 microglia cells, treated with 15 µM of each interest compounds. The authors highlighted that, among the analysed phenylpropionamides, those with a higher inhibitory activity on the Tumor Necrosis Factor α (TNF-α release were lignanamides and compared to resveratrol—the positive control—the much stronger inhibitory action was found for the CLG. To understand the molecular mechanism underlying the neuroprotective action of the most bioactive hempseed phenylpropionamides, various studies were performed by different authors. More specifically, to date, the investigated compounds were CLG [108], grossamide [109], and cannabisin F [110]. In all cases, BV2 microglia cells were pre-treated with 5, 10, or 15 µM of each tested compound 1 h before the addition of 100 ng/mL of LPS for 24 h. After 24 h of LPS exposure, it has been shown that all three lignanamides were able to decrease the release of the inflammatory cytokines IL-1β, IL-6, and TNF-α in a dependent-manner by the inactivation at different level of the NF-κB inflammatory pathway. Moreover, CLG and cannabisin F were also found to have the ability to promote the expression of nuclear factor erythroid 2-related factor 2 (Nfr-2), a protein important in modulating redox homeostasis, in regulating the cellular antioxidant response against reactive oxygen species (ROS) and in attenuating oxidative stress. Normally, this protein is located in the cytoplasm, but in response to oxidative stimuli, Nrf2 transferred to the nucleus and activated downstream genes involved in the antioxidant defence. Although LPS stimulation leads to an increased production of ROS by microglia-stimulated cells, it has been demonstrated that the pre-treatment with CLG or cannabisin F upregulated the Nfr-2 pathway, thus exerting a beneficial antioxidant effect [108,110]. In addition, it has also shown that cannabisin F was able to enhance its inhibitory action on the NF-κB transcription factor even by inhibiting the downregulation of the silent information regulator transcript-1 (SIRT1) induced by LPS. SIRT1 is a histone deacetylase that inhibits NF-κB transcriptional activity through deacetylation of its p65 subunit, thus leading to the reduction of inflammatory cytokines. LPS stimulation induces the downregulation of SIRT1, increasing the inflammatory status, but this can be counteracted by cannabisin F [110]. The anti-neuroinflammatory and neuroprotective effect of hempseed phenols was observed also in vivo on an LPS-induced neuroinflammatory model mice [111]. In particular, the beneficial effects were obtained after three weeks of treatment with 1 g/Kg/day of hempseed extract (concentration of the extract, 233.52 ± 2.50 µg/mg) and consisted of a reduced expression of the inflammatory cytokines IL-1β, IL-6, and TNF-α in the brains and the prevention of LPS-induced damage to the hippocampal nerve cells as well as an improvement in the animal’s learning, memory, and their cognitive function. The phenylpropionamides isolated from hempseed and the literature studies about their biological action are listed in Table 10.

#### 3.2.2. Hempseed Bioactive Peptides

Together with phenolic compounds, other kinds of hempseed’s functional compounds are the bioactive peptides. The evidence of the existence of these peptides has been demonstrated by the findings about the bioactive features observed for the products obtained by the hydrolysis of hempseed proteins. Indeed, it has been shown that, although hempseed proteins have limited bioactive properties, their hydrolysis provides hydrolysates with higher bioactivity [112,113,114], including antioxidant [72,112,113,114,115,116,117,118], antihypertensive [114,119], antiproliferative [72,112], hypocholesterolemic [120,121], anti-inflammatory, and neuroprotective [113,122] effects. This evidence suggests that bioactive peptides are encrypted in the protein native structure and are released during the hydrolysis process. Depending on the hydrolysis conditions (i.e., type of proteases used, period, and time of hydrolysis), hydrolysates with different kinds and efficiency degrees of activity can be obtained. This is due to the fact that hydrolysis conditions can influence the type of obtained peptides, namely the size and the amino acid profile—and hence, the structure—which in turn, influence the activity and the function of peptides. In this context, literature data agree that important factors which affect the bioactivities of the peptides are their molecular weight and amino acid composition. In general, low-molecular weight peptides are more bioactive than the high-molecular weight ones, since they more easily escape enzymatic degradation in the gastrointestinal tract, and therefore, they can be absorbed and introduced as such into the blood stream than larger peptides. Moreover, small peptides are likely more easily able to interact with specific target sites, such as an enzyme’s active site, in comparison to larger peptides. Instead, the peptide’s amino acid composition is fundamental to define its structure and, therefore, to influence the strength and the efficiency of the interaction with the peptide’s target site; in fact, the amino acids composition is the basis of the peptide’s structure-function relationship.

About hempseed protein hydrolysates, different reports [114,115,116,119,122] confirmed that the type and level of proteases used as well as the time of hydrolysis affect the degree of hydrolysis, the amino acids profile, and the molecular weight of the obtained peptides due to the fact that different proteases have different specificities, and therefore, they will produce different types of peptides with different activities. Moreover, these publications demonstrated that, overall, independently to the hydrolysis’ type, hempseed proteins are highly susceptible to proteolysis and most of the liberated bioactive peptides have a molecular weight less than 10 KDa and a high hydrophobicity rate. More specifically, Tang and colleagues [115] and Wang and co-workers [116] observed that hempseed hydrolysates with a higher degree of hydrolysis, a higher amount of hydrophobic amino acids, and a higher surface hydrophobicity possessed higher in vitro antioxidant activity (particularly, the α, α-diphenyl-β-picrylhydrazyl (DPPH) radical scavenging and Fe^2+^ chelating abilities). Their results were consistent with those obtained by Logarušić and colleagues [112], who also found that the obtained hydrolysates showed cytotoxic and antiproliferative activity on HeLa cancer cells and a stimulatory effect on the proliferation of normal HaCaT cells. However, the effective concentration at which the hydrolysates exerted these functional activities depended on the hydrolysis condition, thus highlighting that different hydrolysis methods generate peptides with different structure and, hence, different activity. Similar results were obtained also by Rodriguez-Martin and colleagues [113], who in addition to the in vitro antioxidant activity demonstrated that specific hempseed hydrolysates’ fractions possessed neuroprotective action, being able to down-regulate the expression levels of TNF-α and IL-6 inflammatory cytokines and to increase the mRNA levels of the anti-inflammatory IL-10 gene at the concentration of 100 μg/mL in LPS-stimulated BV2 microglia cells. Furthermore, Malomo and colleagues investigated both in vitro and in vivo hypotensive [119] and in vitro neuroprotective [122] activity of hempseed protein hydrolysates obtained by different hydrolysis methods, highlighting that the hydrolysates’ activity was determined mostly by the type and sequence of peptides’ amino acids rather than by the degree of hydrolysis that is related to the level of the proteases used and the time of the hydrolysis process. Particularly, the authors observed that the hypotensive effect of hempseed protein hydrolysates was due to their angiotensin-converting enzyme (ACE) and renin-inhibitory activity, which seemed to be related to high proline and phenylalanine content, respectively, though also other additional peptides’ structural factors were likely to be involved in the overall functional effect. In the other work [122], the authors showed that hempseed hydrolysates exert an in vitro neuroprotective action through inhibition of the activity of the acetylcholinesterase enzyme (AChE) and observed that the most active hydrolysates were those with the highest negatively charged amino acids amount. Also, arginine was the second most abundant amino acid found in the hydrolysates with higher AChE inhibitory action, and according to the authors, this was probably due to the fact that it is responsible for binding the peripheral anionic site (PAS) of AChE that is believed to be an important enzyme binding site for inhibitors, thus highlighting the importance of the structure-function relationship. Nevertheless, none of these reports that have investigated the bioactive features of hempseed protein hydrolysates elucidated the amino acids profile and structural features of peptides responsible for the investigated functional effects, nor the specific relationship between their function and structure.

In this regard, Aiello and co-workers [121] found that hempseed peptides with a hypocholesterolemic effect contained 8−10 amino acid residues and were characterized by a high hydrophobicity rate, a hydrophobic N-terminus, and a negatively charged C-terminus. These were identified as features essential to interact with 3-hydroxy-3-methyl-glutaryl-coenzyme A reductase (HMGCoAR), the enzyme that mediates the rate-limiting step of the intracellular cholesterol production. These peptides seemed to be generated especially through peptic hydrolysis of hempseed proteins because it has been observed that, in comparison to hydrolysates obtained using other peptidases, the peptic hydrolysate exerted a significantly higher hypocholesterolemic activity on HepG2 cells already at a concentration of 0.25 mg/mL [120]. This hypocholesterolemic effect has been attributed to a statin-like mechanism, since like statins, the peptic hempseed hydrolysate was able to decrease the cholesterol level (1) by acting as direct inhibitor of HMGCoAR and (2) by increasing the level of sterol-responsive element binding protein 2 (SREBP2), a transcription factor playing a crucial role in the regulation of genes involved in the cholesterol homeostasis, such as the low-density lipoprotein receptor (LDLR) coding gene. Therefore, the increase in the SREBP2 level leads to an increase in the LDLR expression on the HepG2 surface and to a consequent increase in the circulating cholesterol uptake.

As said, another bioactive property attributed to hempseed protein hydrolysate was the anti-hypertensive activity [119]. Publications investigating the structural features of the hydrolysate’s component peptides found that most bioactive peptides able to inhibit renin and/or ACE contained three or four amino acids and some particular structural characteristics related to its sequence. They had a good hydrophobicity/hydrophilicity balance; contained hydrophobic amino acids at the C-terminal, aromatic, or branched-chain amino acid residues near the C-terminal; and hydrophobic residues at the N-terminal. Moreover, when a branched-chain, hydrophobic amino acid residue and/or an ionizable functional group were present at the N-terminal or near it, the ACE-inhibitory activity may be enhanced [121,123,124,125]. Hempseed peptides with these structural features that has been shown to possess in vitro ACE-inhibitory activity were the Glycine-Valine-Leucine-Tyrosine (GVLY), Leucine-Glycine-Valine (LGV) [124], Tryptophan-Valine-Tyrosine-Tyrosine (WVYY), and Proline-Serine-Leucine-Proline-Alanine (PSLPA) peptides, whereas the peptides Tryptophan-Tyrosine-Threonine (WYT), Serine-Valine-Tyrosine-Threonine (SVYT), and Isoleucine-Proline-Alanine-Glycine-Valine (IPAGV) showed dual inhibition of ACE and renin enzymes [123], with the tripeptide WYT exhibiting the strongest renin inhibition related to the presence in its sequence of the main structural features characteristics of renin-inhibitor peptides, namely one hydrophobic side chain (tryptophan) at the N-terminal, bulky chains (tyrosine) at the C-terminal, one hydrogen bond donor (tryptophan), and two hydrogen bond acceptors (threonine and tyrosine) [125]. Girgih and colleagues [125] found that the hempseed peptides inhibited ACE through a mixed-type model of enzyme inhibition, implying that peptides bound to both active and non-active sites, thus blocking the substrate binding or producing changes in the enzyme’s conformation, which reduced the substrate affinity with the active site. These interactions between peptide and ACE were mainly stabilized by electrostatic and hydrophobic interactions and hydrogen bond formation. Whereas the hempseed peptides inhibited renin enzyme by an uncompetitive model of enzyme inhibition, implying that peptides bound to the renin−substrate complex, causing reduction in the reaction velocity as well as the catalytic efficiency. Also in this case, the main involved interactions were the electrostatic and hydrophobic ones, as well as multiple hydrogen bonds. Of note, these bioactive peptides originated by simulated gastrointestinal digestion of hempseed proteins. In addition, the in vivo hypotensive ability of the WVYY, PSLPA, WYT, SVYT, and IPAGV peptides were assessed after oral administration of 0.5 mg/mL of each peptide to spontaneously hypertensive rats. The results demonstrated that all analysed peptides were able to significantly reduce the systolic blood pressure (SBP) of animals, but PSLPA, SVYT, and IPAGV exerted a long-lasting SBP-lowering effect, indicating a more efficient absorption coupled with strong binding to target enzymes and resistance to structural inactivation by gastrointestinal tract or blood circulatory system proteases [123].

Hydrophobicity represents a particularly important feature for all bioactive peptides in general and for the antioxidant ones in particular. Indeed, along with the short length of the peptides, hydrophobicity is essential to facilitate the crossing of the intestinal barrier and to enhance the peptides permeability through the cell membrane via hydrophobic association with the lipid bilayer, and in antioxidant peptides, it promotes the effective interaction with free radicals and their inhibition, thus achieving potent antioxidant effects [121]. It has been shown that the hydrophobic amino acids such as valine, alanine, and leucine, represent 50% of the total amino acids in antioxidant hempseed peptides and that they are distributed on both sides of the peptide chain [126]. The hydrophobic, aromatic amino acids such as phenylalanine and tryptophan are also abundant since they can enhance the metal chelating ability due to the several binding sites on their aromatic rings [127]. Another amino acid residue recurring in the sequence of antioxidant peptides, is histidine, especially located at the N- or C-terminal where it can enhance the radical scavenging activity due to its imidazole ring that is effective in metal-ion chelation [126,127].

Finally, Ren and co-workers [128] identified two hempseed peptides with α-glucosidase inhibitory activity and therefore, with antidiabetic property. These peptides were the dipeptide Leucine-Arginine (LR) and the pentapeptide Proline-Leucine-Methionine-Leucine-Proline (PLMLP). Even in this case, the hydrophobic amino acids gave a great contribution to the performance in the inhibition of α-glucosidase activity, and the P and L residues seemed to be particularly important; in fact, they were been considered as vital amino acids playing α-glucosidase inhibitory action separately or synergistically.

In conclusion, it is important to highlight that, although the in vitro and, in part, in vivo (animal) studies have demonstrated the functional effects of hempseed protein hydrolysates and of single isolated peptides, human studies investigating the peptides’ stability into the gastrointestinal tract and their bioavailability, namely the capacity to reach the target site in the active and functional form, are still lacking and they should be investigated.

## 4. Hempseed Dietary Supplementation

Considering the antioxidant and anti-inflammatory properties related to the hempseed’s components, different authors investigated whether hempseed food supplementation could have the ability to counteract chronic-degenerative diseases characterized by chronic inflammatory and oxidative stress conditions, such as cardiovascular and neurodegenerative diseases. Until now, most of the studies have been conducted on animal models and only a few literature reports have been found on humans, underlining the requirement to investigate the effects of the supplementation with hempseeds and their derivatives on human health and to understand their potentiality as ingredient for functional foods. With this in mind, in the following subsections, literature publications about the effects of hempseed- and hempseed products-based supplementation in animals’ and humans’ studies have been reviewed.

### 4.1. Animal Studies

According to the WHO, cardiovascular diseases (CVDs) are the first cause of worldwide death [129]. Atherosclerosis, hypercholesterolemia, and hypertension represent among the major risk factors of CVDs and both hypercholesterolemia and hypertension, along with chronic inflammation and oxidative stress, are considered as the factors that promote the process of atherosclerosis [130]; hence, their prevention would reduce the risk of atherosclerosis and CVDs onset. Diet represents an important non-pharmacological tool that can reduce cardiovascular risk and, thus, improve health outcomes of people at risk, and in this context, the antioxidant and anti-inflammatory properties of whole hempseed could represent an effective and efficient dietary intervention to promote the treatment and prevention of the CVDs. Therefore, some authors investigated the potential anti-hypercholesterolemic, anti-hypertensive, and anti-atherosclerotic ability of hempseeds or hempseed derived products such as hempseed oil, hempseed flour, or hempseed protein hydrolysate, after dietary administration, mainly on rats’, mice’s, or rabbits’ CVDs models.

Regarding this, Kaushal and colleagues [131] evaluated whether 1 month of supplementation of 10% whole hempseeds in Wistar rats fed high-fat, pro-hypercholesterolemic diet had the ability to ameliorate hypercholesterolemia-associated cardiovascular effects. The obtained results highlighted that when 10% whole hempseeds was added to a normal, balanced diet, it did not caused any changes in the serum lipid parameters, the aorta’s wall histology, and the redox and inflammatory status of the animals in comparison to the control (i.e., rats fed a normal, balanced diet without hempseeds supplementation), however, the supplementation of a high fat diet with 10% hempseeds led to a significantly ameliorated effect of serum parameters (i.e., decreased total cholesterol, LDL, and triglycerides levels), redox and inflammatory status, as well as the prevention in the development of the aortic changes related to atherosclerosis. As said, inflammation and oxidative stress are involved in the atherosclerosis changes. It is known that high-fat diet is responsible for the activation of the inflammation inducible genes such as cyclooxygenase-2 (COX-2), which lead to increase the expression of pro-inflammatory prostaglandins Prostaglandin E (PGE). The COX-2 inflammatory pathway is closely related to the redox status because it can be activated by oxidative stress and vice versa (i.e., the COX-2 inflammatory pathway can generate oxidative stress). Based on the obtained results, the authors asserted that 10% hempseeds supplementation was able to exert the anti-hypercholesterolemic effects through redox-sensitive modulation of the inflammatory pathway of PGs biosynthesis. In particular, it would seem that the antioxidant properties of hempseed, which include the ability to scavenge free radicals, to chelate metal ions, and to inhibit the LDL oxidation, may improve the redox status in the high-fat diet induced hypercholesterolemic rats, and thus, it can induce a redox shift of the COX-2 pathway toward the production of anti-inflammatory PGs of D- and J-series.

The beneficial effect of the short-term (20 days) hempseed feeding in Wistar rats on the prevention of hypercholesterolemia and coronary artery disease was observed also by Karimi and co-workers [132]. Indeed, they found that the hempseed dietary treatment did not significantly affect the mean fasting triglyceride, total cholesterol and albumin levels, but it was able to improve the blood lipid and protein profiles, since it significantly decreased the mean fasting serum LDL level, and significantly increased the mean fasting serum HDL and total protein levels. These actions were attributed to the high and low contents of PUFAs and SFAs, respectively, in hempseeds, as well as to their relatively high amount of phytosterols, especially β-sitosterol. Actually, Seo and colleagues [133] showed that also hydrophilic components of hempseeds could exert an anti-atherosclerotic effect. In their study, the authors treated via intragastric inoculation Apo-E Knock Out (KO) mice atherosclerotic animal models, with a hempseed water extract for 14 weeks. The lack of Apo-E in these genetically modified mice led to increase in total plasma cholesterol and triglycerides and decrease of serum HDL-cholesterol level and, as consequence, generated atherosclerotic lesions similar to those found in humans. The treatment with hempseed water extract reduced the atherosclerotic plaque formation, as well as the serum level of total cholesterol and LDL-cholesterol and increased the serum HDL-cholesterol level. Moreover, a reduction in the serum level of the inflammatory cytokine TNF-α demonstrating a decrease in the inflammatory status related to atherosclerosis was found also. As previously said, all these findings have been generally attributed to the hempseed lipidic fraction, that is missing in the hempseed water extract used in this study, and therefore, the authors concluded that some hydrophilic compounds of hempseed, presumably phenolics, possessed the observed anti-atherosclerotic action, but the specific compounds and the molecular mechanisms by which they exerted this protective action have still to be elucidated.

The ability of the dietary hempseeds supplementation to reduce the dietary cholesterol uptake in the presence of an enriched-cholesterol diet, was observed also by Lee and co-workers [134] in *Drosophila melanogaster* and was attributed to the ability of some hempseed components, PUFAs LA and ALA *in primis*, to down-regulate the expression of the Niemann-Pick C1-Like 1 (NPC1L1) gene, coding for a key intestinal cholesterol absorption protein. By contrast, Prociuk and colleagues [135] found that 8 weeks of 10% hempseeds supplementation of a cholesterol-enriched diet in albino New Zealand rabbits did not decrease the cholesterol plasma level but led to an approximately 12-fold increase in the plasma GLA level, resulting in a normalization of the platelet aggregation. Indeed, it was shown that the enriched-cholesterol diet without hempseeds supplementation increased the cholesterol plasma level and the platelet aggregation, raising the risk of acute coronary syndrome. The addition of hempseeds to the diet was however able to inhibit the platelet aggregation despite the presence of high circulating cholesterol, and this effect was exactly attributed to the high GLA plasma level. All these results that showed an overall beneficial effect of hempseeds dietary supplementation on atherosclerosis are still in contrast to those found by Gavel and co-workers [136]. Indeed, they found, by investigating the effect of the addition of 10% hempseeds to the cholesterol-enriched diet of albino New Zealand rabbits on atherogenesis and aortic contractile function, that this dietary supplementation would not be able to protect the aortic vessel from the atherosclerotic plaque formation. In this case, the hempseeds supplementation provided only mildly beneficial effects against contractile disfunction associated with atherosclerotic vessel.

In female subjects, the risk of coronary heart disease and other CVDs related to the rise of the concentration of circulating total cholesterol and LDL-cholesterol, can also increase as a consequence of menopause, which is associated also to other relative complications related to the estrogen deficiency, like depression, anxiety, and possibly impaired cognition. In this context, Saberivand and colleagues [137] demonstrated that whole hempseeds dietary supplementation (until 10%) in a balanced diet of the ovariectomized rat model of menopause, may improve the menopausal complications including the cardiovascular ones. In particular, the authors have shown that 3 weeks of dietary supplementations with 1%, 2%, or 10% of hempseeds were able to conserve the triglycerides and total cholesterol plasma level close to the control group (non-ovariectomized rats), preventing the dyslipidemia and the weight gain that usually occurs after ovariectomy (or menopause) as a consequence of the decline of the estrogen levels. Indeed, it was found that the decline of plasma estrogen levels was prevented by the hempseeds dietary treatment, probably thanks to the presence of phytoestrogens, such as β-sytosterol, in hempseeds. In addition, the authors even observed that the hempseeds dietary supplementation was also capable of exerting an antidepressant activity, as well as of preventing the accrual of blood calcium and thus preventing the bone loss and promoting the bone homeostasis.

As previously underlined, along with hypercholesterolemia, hypertension represent another main CVDs risk factor. It is defined as a systolic blood pressure (SBP) higher than 140 mmHg or a diastolic blood pressure (DBP) higher than 90 mmHg [138]. The in vitro studies which demonstrated the existence of hempseed peptides with anti-hypertensive properties (see Section 3.2.2) led some researchers to evaluate the in vivo hypotensive ability of hempseed products on the spontaneously hypertensive rats (SHRs). This animal model is characterized by high plasma ACE and renin enzyme activities as well as a decrease in the antioxidant defence and higher oxidative stress in comparison to the normotensive rats. It has been shown that a diet supplemented with 1% of hempseed proteins hydrolysate was effective both to prevent the hypertension development in SHRs and to act as treatment to reduce the SBP in SHRs with still established hypertension through the reduction of the plasma ACE activity [138]. Moreover, the same supplemented diet led also to a reduction of the oxidative stress in SHRs normalizing the plasma total antioxidant capacity, which reached a value similar to normotensive rats, and enhancing the cellular antioxidant defences, namely the catalase and superoxide dismutase enzymes’ activity [117]. The anti-hypertensive and antioxidant effects were not so marked in the SHRs fed a diet supplemented with 1% hempseed protein isolate instead of the hempseed protein hydrolysate. This evidence was explained with the fact that the already hydrolysed peptides are more bioactive or more bioavailable than those obtained by the gastrointestinal digestion of hempseed proteins [117,138], suggesting the advantage of the use of hempseed protein hydrolysate rather than whole hempseed or hempseed proteins isolate as an ingredient to formulate functional foods or nutraceuticals for the prevention and treatment of hypertension. Interestingly, the hypotensive effect of these hempseed products was not observed in normotensive rats, suggesting an action directly related to the higher ACE plasma activity than the physiological level [138].

Others animal studies were performed to explore the potential beneficial effect of the dietary intake of hempseeds and their products on neurodegenerative diseases, generally associating with aging, oxidative stress, and neuroinflammation, such as AD, cognitive deficit, memory impairments and multiple sclerosis (MS). Most of these studies were carried out after that epidemiological evidence highlighted that some Chinese population, habitual consumers of hempseeds which make up an integral part of their daily diet, are characterized by significant numbers of long-lived centenarians’ individuals with good cognition and few health problems [139,140]. About this, Lee and co-workers [134] found that a *Drosophila melanogaster* model of AD reared in a hempseed meal media, significantly suppressed the cytotoxicity of amyloid β42 (Aβ42) peptides accumulation, ameliorating the eye degenerative phenotype, and this neuroprotective effect of hempseed meal was attributed to the PUFAs LA and ALA. Further evidence has demonstrated that the daily inclusion and long-term consumption of hempseeds into the diet may promote the longevity and the lifespan extension; may improve cognitive function; and may prevent age-related diseases, including obesity, diabetes, and cancer in old mice, as a consequence of the adjustment of some nutritional imbalances typical of the western diet (e.g., the n-6/n-3 PUFAs ratio), and the capability to regulate the expression of genes involved in the regulation of 5′AMP-activated protein kinase (AMPK) signalling that can reduce the oxidative stress and can inhibit the inflammatory response induced by the NF-κB pathway [140]. It has been also shown that daily oral administration of hempseed oil on an MS mice model can have positive effects on the treatment of this chronic-inflammatory and neurodegenerative disease characterized by wide demyelination, since it can exert immunomodulatory action, in particular the anti-inflammatory one, and can improve the re-myelination of neuronal myelin sheath in the brain, with a protective effect on the central nervous system (CNS) [141]. This protective effect of hempseed oil in CNS tissues seems to be a consequence of the increase in the intake of n-3 PUFAs and the n-6 anti-inflammatory GLA and of the reduction of the n-6/n-3 PUFAs ratio and of SFAs intake, all of which are factors able to regulate the mTOR signalling pathway in order to shift the immune response from pro- to anti-inflammatory one, thus reducing the chronic inflammation, which is an important factor for MS onset and development [141,142]. However, in many of the reports that demonstrated the neuroprotective effect of hempseeds or derivatives dietary supplementation, the hempseeds’ supplement was not the only one administrated. In fact, hempseeds were often administrated in combination with other products such as bitter vegetable (*Sonchus oleraceus*) [140] or evening primrose oil [141,142], and therefore, based only on these data, it is not possible to understand whether the beneficial effects can be obtained also with the solely ingestion of the hempseeds supplement or whether they are closely related to a co-operative action of the both administrated supplements.

Hence, although different evidence of an effective beneficial action of hempseeds or hempseed derivatives on CVDs and neurodegenerative disease animal models is present in scientific literature (Table 11), further studies are still needed to clarify the exact molecular mechanisms involved as well the type and the doses of the more efficient hempseed product necessary to exert the in vivo beneficial function, that are all essential factors to develop a potentially functional food.

### 4.2. Human Studies

Although the studies about hempseeds and hempseed products dietary supplementation in animals are relatively abundant in scientific literature, only a few papers (Table 12) have faced the issue of the hempseeds and derivatives dietary supplementation in human nutrition and its effects on human health, and all of the few investigations performed to date, were carried out exclusively with hempseed oil as a supplement.

One of the first studies which investigated the beneficial effect of hempseed oil dietary supplementation was that of Callaway and co-workers [143] in which a 8-week oral consumption of 30 mL/day of hempseed oil in patients with diagnosis of atopic dermatitis, was adopted to evaluate the potential ability of this oil to ameliorate the patients’ symptomatology and medicinal usage. The results of this research highlighted that, contrarily to the olive oil treatment, used as comparison, the hempseed dietary assumption can effectively improve the skin quality and the pathology’s symptoms leading to decrease of skin dryness, itchiness, and dermal medication usage by patients. The authors attributed these findings to the increase in the GLA, SDA, and LA plasma levels as a result of the hempseed oil supplementation. Indeed, both GLA and SDA have anti-inflammatory action which can be beneficial for atopic dermatitis. Particularly, GLA has been shown able to decrease the production of the pro-inflammatory leukotriene B4 (LTB4) by polymorphonuclear neutrophils (PMN), through increasing the plasma level of its biological metabolite, namely the anti-inflammatory DGLA which can directly suppress the LTB4 PMN’s production in the skin. Furthermore, SDA can increase the level of EPA in both erythrocytes and plasma phospholipids contributing to the anti-inflammatory effect, whereas, the increase of the LA plasma level can raise the ceramide amount in the skin’s stratum corneum, reducing the loss of moisture and improving the skin’s dryness, since LA acts as precursor of ceramide 1, an important component of the skin’s lipid lamellar sheet. Finally, the total plasma PUFAs amount of the patients significantly increased after the oral daily assumption of hempseed oil, and this can have facilitated the formation of new skin cells by dermal stem cells, contributing to the obtained amelioration of atopic dermatitis.

Other works employed the hempseed oil as a dietary treatment/supplementation to explore its potential cardiovascular benefits on humans. In this regard, Schwab and colleagues [144] observed that 4-week daily supplementation of 30 mL of hempseed oil was able to reduce the total TGs plasma level and to induce significant changes in the composition of serum cholesteryl-esters (CEs) and TGs of healthy volunteers. Particularly, the content of LA, ALA, and GLA increased whilst the OA concentration significantly decreased after the hempseed oil treatment. In contrast, the proportion of AA and DHA in both TGs and CEs did not change after hempseed oil intake as well as the plasma glucose, insulin, and haemostatic factors, including fibrinogen, coagulation factor VIIa (FVIIa), and plasminogen activator inhibitor-1 (PAI-1). Interestingly, the hempseed oil treatment led to reduction of the TC/HDL-cholesterol ratio which is considered an important index to predict the risk of coronary heart disease. Contrarily, Kaul and colleagues [145] found that the daily assumption of 2 capsules, each containing 1 g of hempseed oil, in healthy volunteers for 12 weeks neither significantly affected any blood lipid parameters (i.e., the TGs, TC, LDL, and HDL plasma levels), nor induced any changes in the LA and ALA plasma concentrations as well as in the inflammatory and platelet aggregation markers, namely TNF-α and C-Reactive Protein (CRP), and collagen and thrombin-induced platelet aggregation, respectively. The authors hypothesized that these findings could be due to a limited and low gastrointestinal absorption of hempseed oil’s LA and ALA, or to the fact that a larger dosage and a longer experimental period may be necessary to obtain significant changes in the evaluated parameters. It is also important to note that these studies have been performed on healthy subjects, and therefore, they do not exclude potential beneficial actions in a specific pathological status. Furthermore, this evidence also introduces the importance and the necessity of identifying the appropriate dosage and vehicle/matrix that will have benefits in healthy people too as a preventive tool. In the context of the usage of hempseeds or their products for a specific pathological target groups, a recent report of Del Bo’ and colleagues [146] explored the effects of hempseed oil supplementation in the modulation of hyperlipidaemia and CVDs risk in children and adolescents (6–16 years old) with primary hyperlipidaemia. In more detailed, the test group was daily supplemented with one hempseed oil soft capsule containing 3 g of oil, which provided 700 mg of ALA, 1400 mg of LA, 30 mg of SDA, and 100 mg of GLA, with 2.3 g total PUFAs and a PUFAs/SFAs ratio of 7.7:1. As a consequence of this treatment, it has been shown that, although the hempseed oil supplementation after 8 weeks did not significantly change the plasma lipid profile of the volunteers, it was able to improve the red blood cell (RBC) FAs composition, significantly increasing the total n-3 PUFAs, including the LC-n-3 PUFAs EPA, DPA, and DHA and decreasing the n-6/n-3 PUFAs ratio, as well as the total amount of SFAs and MUFAs. Considering that an altered FAs composition in RBCs has been shown to be positively related with coronary heart disease, atherosclerosis-related diseases, arterial hypertension, and dyslipidaemia, and that SFAs have been reported to have hypercholesterolemic activity in adults, the observed effect of hempseed oil supplementation could have a contribution in counteracting the negative consequences of hyperlipidaemia on the cardiovascular system. Interestingly, in this work was also noted that the dietary intake of higher ALA amounts led to an increase in the RBCs’ LC-n-3 PUFAs derived by the ALA conversion (EPA, DPA, and DHA) in comparison to the control group, and this evidence could indicate that hempseed oil supplementation may stimulate the endogenous conversion of ALA into its biologically active derivatives. According to the authors and in agreement with Kaul and colleagues [145], the lack of differences between the control and the test group on the serum lipid markers, may be dependent on the food matrix/vehicle used and/or the length of the intervention. This evidence has highlighted again the importance to understand the molecular mechanisms through which hempseed components can exert their beneficial effect on human health, the level of each functional component needed in order to obtained an effective and clinically detectable action, and the more suitable matrix able to maximize the gastrointestinal bioavailability and structurally preservation of these components in the human body.

Finally, another three literature works about the hempseed oil dietary supplementation in humans have been performed on patients affected by MS [147,148,149]. As described in the previous subsection (Section 4.1), the same authors have used an MS mouse model in order to understand the molecular mechanisms underlying the beneficial effects of the co-supplementation of hempseed oil and evening primrose oil on this neurodegenerative, immune-mediated pathology [141,142]. In previous works performed on humans, these authors observed that the co-supplementation of hempseed oil and evening primrose oil in a 9:1 ratio, used alone, or in association to a Hot-natured diet, was able to ameliorate the inflammatory status of MS patients, thus exerting a beneficial effect on this pathological condition. In particular, the authors explained that MS is a Th1-mediated autoimmune disease, which therefore, is characterized by an unbalance Th1/Th2 immune responses and high levels of pro-inflammatory IL-6, IFN-γ and IL-17 cytokines. This latter cytokine is produced by the Th17 cells which, hence, are involved in the development of the pathology, together with Th1 cells [147,148]. A Hot-natured diet is characterized by the consumption of foods with low cholesterol, hydrogenated, *trans*, and saturated FAs; a plenty of fruits and vegetables, nuts and seeds without additives, olive and grapes oils as the main oils of the diet, fish and seafood, and unrefined carbohydrates and dairy products with honey; contrarily, it excludes the consumption of sugar and refined carbohydrates, alcohol, and smoke. It has been shown that the consumption of the Hot-natured diet is related to a deviation of the immune system toward the Th2 responses. In addition, both hempseed oil and evening primrose oil possess anti-inflammatory features, related to the high PUFAs content and the perfectly balanced n-6/n-3 PUFAs ratio of the former and to the about 9% of GLA content of the latter. Based on all this evidence, Rezapour-Firouzi and co-workers in their studies [147,148] decided to adopt the hot-natured diet co-supplemented with 18–21 g of hempseed oil and evening primrose oil in a 9:1 ratio, as a potential useful dietary intervention for the treatment and attenuation of MS. The authors found that both types of dietary intervention (i.e., the Hot-natured co-supplemented with hempseed oil and evening primrose oil, and the co-supplementation of hempseed oil and evening primrose oil alone) for 6 months have exerted an effective anti-inflammatory action, leading to a significant increase in the IL-4 cytokine level on MS patients and to an improvements of the degree of the Th2/Th1 ratio, the expanded disability status scale (EDSS), and the relapse rate, which are all indexes of the disease severity, and thus performed a beneficial action in the patients, without exhibiting any side effects [147,148,149]. However, only the Hot-diet associated with the hempseed and evening primrose oils co-supplementation resulted in having also the ability to significantly decreased the level of the pro-inflammatory IFN-γ and IL-17 cytokines. Indeed, contrarily, the hempseed oil and evening primrose oil co-supplementation alone showed a decreased trend in the level of these cytokines, which though, was not statistically significant, at least after 6 months of the treatment [147]. In a later report, the same authors [149] also demonstrated that both Hot-natured diet co-supplemented with hempseed and evening primrose oils and the co-supplementation of hempseed and evening primrose oils alone were able to improve the liver function in the MS patients, which generally showed a serious liver injury also as a consequence of the drug treatment, but the former dietary intervention was more efficient than the latter.

In conclusion, in addition to the fact that there is still a lack of studies about the effects of whole hempseed dietary intake on either healthy human subjects or patients with a specific pathological status, to date, the few works present in the scientific literature refer exclusively to hempseed oil supplementation and they are rather inconclusive and not very comparable due to the employment of different types of experimental designs, as well as different dosages, experimental periods, and administration methods of the supplement. Therefore, it appears necessary to deepen and expand the research and the knowledge in this area.

## 5. Hempseed Foods

In the field of food technology, due to their unique nutritional quality and composition, hempseeds have been exploited as ingredient or means to ameliorate the nutritional and healthy grade of food, in order to obtain healthy and potentially functional foods. In this context, the first efforts in the employment of these seeds by the food industry concerned their use as a means to increase the quality of animal derived food products, namely the use of hempseed or hempseed’s derivatives (i.e., hempseed oil or flour/cake/meal) as livestock feed supplement to ameliorate the quality composition of animal-based food products (e.g., meat, eggs, and milk). Only later, hempseeds and their derivatives have also been used as ingredient to directly enrich daily-consumed foods (e.g., bakery products).

### 5.1. Hempseed Products as Livestock Supplement

As a means to increase the quality of animal-derived food products, hempseeds and hempseed derivatives, especially hempseed oil, were used as supplements of the laying hens or broiler chickens’ normal diets to obtain eggs or meat enriched with n-3 PUFAs, including the n-3 long chain PUFAs (LCPUFAs) biologically active in humans, like EPA and DHA, due to the ability of the laying hens or broiler chickens to endogenously convert dietary ALA into these LCPUFAs and to deposit them into egg yolk or muscle tissues. In this context, several experiments were performed to assess either the safety of hempseed or hempseed derivatives as feed ingredients in poultry diets as well as their efficacy as a means to nutritionally ameliorate the FAs profile of egg yolk and meat, as an alternative to other n-3 PUFAs sources such as flaxseed, flaxseed oils, or marine oils, and microalgae products. Overall, these studies demonstrated that the performance of laying hens and broiling chickens, including feed intake, egg weight, egg production, feed conversion ratio, body weight, or weight gain, was not affected [150,151,152,153] or was ameliorated [154] by the dietary supplementation with hempseeds (up to 30%) or hempseed oil (up to 12%), and therefore, these products can be included in the bird’s diet without compromising their safety and efficacy. Interestingly, the hens’ performance was more positive if hempseeds were subjected to a heat treatment (specifically 120 °C for 60 min) performed before to supplement the hens’ diet [155]. The safety of the inclusion of these supplementary products to poultry diet was further confirmed by the absence of the markers of hepatic and muscular damage after the hempseed product supplementation [151,154]. In addition, literature reports agree that the inclusion of hempseed products to the poultry diet effectively improved the nutritional value of the eggs and meat and obtained food products with higher total n-3 PUFAs amount, higher PUFA/SFA ratio, and a better-balanced n-6/n-3 PUFAs ratio in comparison to the poultry food products obtained by animals fed a normal diet. More specifically, it has been demonstrated that the total n-3 PUFAs and the individual n-3 PUFA ALA, as well as the n-3 LCPUFAs biologically active in humans, namely EPA, DPA (Docosapentaenoic Acid), and DHA, were increased in both poultry eggs [150,152,153,156,157,158,159,160] and meat (thigh and breast) [150] as the inclusion of the hemp products rose and the n-6/n-3 PUFAs ratio, normally high in common eggs, was reduced [152,157,158,161]. Neijat and colleagues [157] found that, as a consequence of the dietary hempseed and hempseed oil supplementation to the laying hens’ control diet (wheat-barley-soybean meal-based), the ALA content increased in a dose-dependent manner and this increase was higher in the TGs than in the phospholipids (PLs) that, therefore, seem to be less responsive in ALA enrichment in comparison to TGs. The authors found also an increase in the total amount of n-3 LCPUFAs synthesized from ALA (i.e., EPA, DPA, and DHA) in response to the increase in the ALA dietary intakes, reflecting the endogenous synthesis from ALA in hens’ liver and the subsequent deposition in egg yolk. However, contrarily to ALA, this growth did not occur in a dose-dependent fashion for LCPUFAs, such as DHA, which in fact, was accumulated mainly in PLs, until a saturation level [156,157]. Regarding this, it is important to consider that the conversion of ALA to DHA occurs through the sequential actions of the enzymes ∆-6 desaturase encoded by the fatty acid desaturase 2 (FADS2) gene, elongase encoded by the elongase 5 (ELOVL5) gene, and ∆-5 desaturase encoded by the fatty acid desaturase 1 (FADS1) gene. Gakhar and co-workers [152] by evaluating the effect of the consumption of hempseeds or hempseed oil by hens on the expression of these three main biosynthetic genes involved in the PUFAs metabolism found that the FADS1 and FADS2 expressions were reduced by the increasing inclusion of hempseed products in laying hens’ diets, thus providing a possible explanation to the DHA saturation in the egg yolk of hens supplemented with hempseed products. Interestingly, Jing and colleagues [150] showed that the increase in the n-3 PUFAs ALA, EPA, DPA, and DHA occurred both in the egg yolk and in the meat of laying hens’ and broiler chickens fed a hempseed oil supplemented diet, but this increase took place to a different extent based on the food product. Indeed, in comparison to the respective control, the enrichment efficiency for total n-3 PUFAs, ALA, and EPA appeared to be higher in egg yolk than in chicken meat. Furthermore, the DPA amount was higher than DHA in meat, contrarily to egg yolk, where the DHA level was higher than DPA even without supplementation, and after hempseed oil supplementation, the difference between DPA and DHA content in egg yolk or meat became more evident. This finding was attributed to a differential expression and activity of FADS2 and ELOVL5 enzymes that are involved in the conversion of DPA to DHA rather than an effect of the supplementation. In particular, based on the results, it was hypothesized that these enzymes may be low in the muscle tissues of chicken and thus, inhibiting the metabolism of DPA to DHA. Interestingly, Mierliță and colleagues [153] found that the egg yolk produced by hens fed a diet supplemented with whole hempseeds contained a higher ALA and EPA amount and a more remarkable decrease in n-6/n-3 PUFAs ratio in comparison with the egg yolk produced by hens fed a diet supplemented with hempseed cake. Hence, the authors concluded that whole hempseeds supplementation was more effective in ameliorating the nutritional quality of egg yolk than the hempseed cake one.

With regard to the effect of hempseed products supplementation on the n-6 PUFAs content of the poultry eggs and meat, Shahid and co-workers [160] found an increase in the total n-6 PUFAs amount of egg yolk, whereas, Neijat and others [157] observed that, although the level of n-6 LA was higher especially in the TGs, in comparison to the control, the n-6 AA amount decreased as the hemp dietary inclusion increased, reflecting the metabolic competition of n-3 ALA and n-6 LA for the ∆-6 desaturase enzyme, responsible to their conversion in the corresponding LCPUFAs, with the greater affinity of n-3 ALA than n-6 LA, and a consequent favour of n-3 PUFAs metabolism. Similar data was found also by Mierliță and colleagues [153]. Furthermore, it has been also observed that among, n-6 PUFAs, the hempseed products supplementation led to an enrichment of eggs and meat with the GLA [150,161], conferring to these food products a further unique features related to the biological anti-inflammatory and anti-proliferative properties of GLA that have important implications for human health. Similar GLA-enrichment was also shown in the muscle of ducks fed a 15-20% hempseed cake-supplemented diet [162].

Regarding MUFAs and SFAs, literature publications agree that the MUFAs content, mainly OA, decreased with the increase of the level of the hempseed products to the diet, probably due to the inhibition effect of a PUFAs-rich diet on the activity of ∆-9 desaturase, which is essential for the conversion of PA to OA [150,153,155,157]. Whereas, about the effect on SFAs there are some contrasting results. Some authors found a reduction in the SFAs [155,158], as well as in the cholesterol amount [161] in egg yolk after hempseed supplementation. Other researchers [153,157] showed that the overall levels of SFAs and cholesterol of egg yolk were not influenced by a dietary treatment with hempseeds, hempseed oil, or hempseed cake.

Similar findings about the positive effect of the hempseed or derivative dietary supplementation on the nutritional quality of egg yolk and meat were also shown for quails [163]. In this case, 6-week supplementation with up to 20% whole hempseeds, allowed to obtain n-3 PUFAs enriched quail eggs with a reduction of MUFAs—OA in particular—and SFAs—PA in particular—as an additional advantage. In the same study, the quality of the meat of the 5-week whole hempseed supplemented quails was also analysed, and a decrease in thawing and cooking loss were observed, which are both negative indicators for meat quality, with the increase of the dietary hempseed amount, demonstrating that hempseeds supplementation can ameliorate also the meat quality other than the eggs FA profile. In addition to the safety for animals and the efficacy in obtaining more nutritionally beneficial food products, the inclusion of hempseed products into the poultry diet was tested also for the potential negative impacts on the sensory characteristics of the resultant products. Indeed, it was found that dietary supplement as flaxseed or fish oils can compromise the sensory quality of the poultry meat and eggs due to the presence of fish smell/taste. Instead, Goldberg and colleagues [159] demonstrated that the inclusion of up to 20% hempseed or up to 10% hempseed oil in the poultry’s diet did not have any adverse effects on the eggs’ sensory quality. Similar results were obtained by Konca and co-workers [155] for eggs produced by hens supplemented with 15% raw or heat-treated whole hempseeds for 12 weeks.

Hempseeds supplementation in animals’ feed has been shown to be a useful tool also to improve the yield, the quality, and the nutritional value of some milks, such as the goat and sheep ones, and in particular, to positively modify the milk’s FAs content. This by increasing the total concentration of LCPUFAs, n-3 PUFAs, and conjugated linoleic acids (CLAs), and concomitantly by decreasing the concentration of SFAs and the n-6/n-3 PUFAs ratio, with a putative beneficial effect on human health [164,165,166]. Mierliță and colleagues [167] found also an increase in the tocopherol amount and an improvement of total antioxidant capacity of milk obtained from sheep fed a diet supplemented with hempseed (180 g/day) or hempseed cake (480 g/day), in comparison to that obtained by sheep fed a control diet (hay diet completed with high-energy concentrate and sunflower meal), thus demonstrating that despite the increase of the PUFAs content in milk, the safety of this product was not negatively affected by lipid oxidation. The same authors also showed that, between hempseeds and hempseed cake supplementation, the latter was more effective in ameliorating the quality and the nutritional value of the milk, as it was resulted in a significantly lower atherogenic index and a higher hypocholesterolemic/hypercholesterolemic index. Cozma and colleagues [168] found a reduction in the lactose content of the milk produced by goat fed a diet supplemented with hempseed oil (93 g/day). Contrarily, Iannaccone and co-workers [165] showed an up-regulation in the genes involved in lactose biosynthesis, based on the results of a RNA sequencing analysis of the whole-blood transcriptome of lactating sheep fed either a control diet or a 5% hempseed supplemented diet, and this result was in accordance with the increase in the lactose amount found in the resulting milk.

### 5.2. Hempseed Products as Ingredient for Enriched/Fortified Foods

As a means to directly enrich or fortify daily foods, hempseeds and their derivatives have been assessed as an ingredient added to daily consumed foods, including bakery products such as bread, cookies, crackers, and energy bars, as well as meat and meat products.

Regarding the enrichment of bread with hempseed or derivatives, some works have evaluated the impact on the nutritional, structural, and textural characteristics of both dough and bread, consequentially to the addition of hempseeds or their derivatives. About this, from a technological/rheological point of view, the main issue of the usage of non-conventional ingredient in the bakery industry, in order to fortify the bakery products is their adverse effect on the dough rheological and baking properties, mainly as a result of the dilution of wheat gluten proteins. Consequently, this can negatively affect the end product quality (volume, texture, crumb and crust properties, and colour) that may ultimately affect lowering the consumers’ acceptability. Studies about the enrichment of the bread dough with hempseed flour agree that the addition of hempseed flour in the wheat flour significantly reduced the dough’s water absorption, stability, strength, and starch gelatinization, in a proportional manner to the level of hemp flour added as a consequence of the dilution of gluten amount in the dough [166,169,170]. Pojić and colleagues [166], and Švec and others [169] found that the decrease in the water absorption of the bread dough had a negative impact also on the time required for dough development, but nevertheless, the dough’s stability and strength were not significantly affected by the addition of up to 10% of hempseed flour. Pojic and others [166] also noted that the increase in the hempseed flour content up to 20%, negatively affected both the dough’s stability and strength.

The changes in the dough’s rheological properties, resulting in the addition of hempseed flour, led to a negative impact on the bread quality. In this regard, Pojic and colleagues [166] showed that the bread volume was significantly impaired as the level of hemp flour increased. This result was found also by Hrušková and co-workers [171] and by Švec and colleagues [169] for bread enriched with 10%, 15%, or 20% of hempseed flour, and was confirmed also by Mikulec and others [172] for bread containing 15%, 30%, or 50% of hempseed flour, as well as by Wang and colleagues [173] for bread enriched with 5%, 10%, or 15% extruded hemp-rice flour (EHR), made by the extrusion of the whole hempseeds (30%) together with the rice flour (70%). According to the authors, the decrease in the bread’s volume could be due to the partial replacement of wheat flour with a non-glutinous one, that may dilute the gluten protein of wheat flour and prevent its extension, or to the increased content of fibre, which reduces the ability to retain fermentation gases. However, Wang and colleagues [173] showed that the addition of 15% of EHR led to a significantly increase of the bread volume in comparison to 10% EHR-containing bread. The researchers attributed this evidence to the higher fat content present in the 15% of EHR. This hypothesis was later confirmed by another work of the same authors [174] in which the properties of the dough and the bread made from extruded and non-extruded hemp-rice flour were compared. The obtained results showed that the presence of whole hempseeds in the bread dough did not impair the specific bread volume in comparison with the control. A similar finding was noted also by Švec and co-workers [169] who found that the volume of the bread enriched with whole hempseeds powder (i.e., ground whole hempseeds) was higher than those obtained for the breads enriched with other defatted hempseed products (i.e., hempseed flour or hempseed protein concentrates). This fact has been attributed to the interactions between lipids and proteins and to the influence of the fat in terms of enhancing dough expansion during proofing [166]. The addition of hempseed flour to the wheat flour also changed the physical and rheological properties of the bread’s crust and crumb. The color of both of them became darker after the hempseed flour addition [166,169,172,173]. Švec and colleagues [169] asserted that the 5% enrichment has conferred a pleasant coffee brown colour, which intensified to dark brown by increasing the amount of hempseed flour, whereas, Wang and co-workers [173] observed a significantly decreased in the lightness of both crust and crumb only for the breads enriched with an EHR amount of at least 10%, since the 5%-EHR bread did not show any significant colour’s differences compared to the control bread. Similar changes in the crust and crumb bread’s colour were observed by Wang and colleagues [174] also after the addition of whole hempseeds powder. Interestingly, in this paper, the authors noted that the hempseed extrusion process and the extrusion temperature can further emphasize the lack of the lightness. Regarding the textural properties of bread crumb, Pojic and co-workers [166] found that the presence of hempseed flour as an ingredient of the bread’s mixture significantly decreased the crumb cohesiveness and increased the crumb chewiness and hardness. Analogously, Mikulec and others [172] found an increase in the hardness of the crumb after the addition of hempseed flour, on the baking day. Contrarily, Wang and colleagues [173] showed that the 15% EHR-bread displayed significantly lower hardness compared to the samples enriched with a lower EHR amount (i.e., 5% and 10%), whereas, in their later publications [172], the same authors observed no significantly differences on the crumb hardness in the whole hempseeds powder-containing breads, and this might be attributed to the influence of hemp oil, which probably enhanced crumb texture and crust tenderness. Interestingly, Pojic and colleagues [166] found that the presence of hempseed flour up to 10% in bread formulation did not significantly affect the elasticity and the resilience of the crumb, unlike the addition of 20% of hempseed flour.

Concerning the nutritional quality of the hempseed flour or whole hempseed powder-enriched bread, it has been demonstrated that the addition of hempseed product significantly ameliorated the bread nutritional quality, since it proportionally increased the total protein [166,169,170,171,172] and fat [166] contents, as well as the total, soluble, and insoluble dietary fibre [169]. About this, it is interesting to note that Švec and colleagues [169] showed that the increase in the dietary fibre was higher if the enrichment was made with whole hempseed powder rather than hempseed flour. Furthermore, Pojic and co-workers [166] found that the hempseed flour enrichment also led to an increase in the content of the macro-elements, especially of Mg and Ca and of the micro-elements such as Mn, Cu, and above all, Fe and Zn. At the same time, the Na amount, the complex carbohydrates, and total metabolizable energy of the bread significantly decreased. This nutritional improvement was more marked for the 20% hempseed flour-enriched bread. Also, an increase in the total phenolic content was observed by Mikulec and others [172] as a consequence of adding the hempseed flour, and the most abundant polyphenols included epicatechin, ferulic acid, and protocatechuic acid. However, according to Švec and co-workers [169], the addition of 20% of hemp product (i.e., hempseed flour or whole hempseeds powder) has conferred a less acceptable, partially bitter taste to the bread; by contrast, the addition of 10% hempseed product did not have any negative impact on the sensorial parameters. Mikulec and co-workers [172] found that also the 15% addition level of hempseed flour had an overall acceptability. Therefore, also considering that the negative impact of the presence of the hempseed flour on the bread quality seems to be attenuated with 10% enrichment, the literature generally agrees that the 10% or 15%-substitution level of hempseed flour should accomplish requirements in terms of providing the balance between bread quality, sensorial, and nutritional properties.

Since hempseed is naturally gluten-free, it could be potentially employed as an ingredient to improve the poor nutritional value of the gluten-free products. To date, only a few papers in the literature investigated this issue by evaluating the impact of replacing to a certain extent, the main ingredient of the gluten-free recipe with hempseed derivatives, mainly hempseed flour or proteins. For example, Korus and colleagues assessed the effects of replacing 10% or 20% of gluten-free starch corn with hempseed proteins or flour in the dough of gluten-free bread [175], and the consequences of the substitution with 20%, 40%, or 60% of hempseed flour in the preparation of gluten-free cookies [176] on the rheological, qualitative, sensorial, and nutritional properties of the relative food products. Nutritionally, both studies highlighted that the addition of hempseed product led to significantly improvement of the nutritional value of the gluten-free food, since it rose, at high extent, the amount of total proteins and total dietary fibre, including soluble and insoluble, with higher increase especially in the insoluble fraction as well as fat and minerals [175,176]. Moreover, for the biscuits, it has shown that the carbohydrate content and the energy value of the products decreased proportionally to the level of substitution. Furthermore, the authors also found an enrichment in the antioxidant profile of the hempseed flour-substituted cookies that had significantly higher values of total phenolic (up to 2-fold higher) and flavonoid (up to 2-fold higher) compounds compared to the control, with a resulting rise in the total antioxidant capacity (up to 3-fold higher) [176]. Hence, from a nutritional point of view, either hempseed flour or proteins may be considered valuable ingredients for the formulation of improved gluten-free products. Regarding the rheological properties of the bread’s and cookies’ dough, the authors showed that for the bread mixture, the substitution of corn starch with hempseed flour resulted in a weakening of dough structure, which became more susceptible to deformation; contrarily, the substitution with hempseed proteins reinforced the dough structure. These differences have been attributed to the differences in the interaction and the binding between the hempseed components and water in the dough. Indeed, the authors explained that proteins absorbed more water, and hence, the addition of hempseed proteins could prevent the fulfil hydration of all dough component, thus resulting in dough strengthening. By contrast, hempseed flour contains other components besides proteins, and these significantly modified water binding, thus differently influencing the rheological properties of final dough [175]. For the cookies’ dough, the authors observed a significant increase in the overall hydration properties of the mixture, demonstrated by the higher values of water absorption, water retention, and oil absorption capacity in comparison to the control. These findings were associated to the increase of both the protein (4-fold higher) and total fibre content (20-fold higher) in the dough, which significantly affected the hydration properties of the biscuits’ blend [176]. In addition, contrary to what was observed for the enrichment with hempseed flour of gluten-containing bread, in this case, the addition of hempseed flour led to a significant increase in the quality of the gluten-free bread, as well as an improvement in the bread sensory acceptability, nutritional quality, and storage. Particularly, the authors found an increase in the bread volume, resulting in the addition of both hempseed products. This increase was more evident with raising amounts of hempseed products in the dough, and it was more pronounced in the case of hempseed proteins addition. This is probably due to the absence of gluten proteins, and so, hempseed proteins can act as surface-active and foaming agents, thus allowing to retain and stabilize the gas bubbles in the dough and bread. Interestingly, the bread volume was shown to be negatively related to the crumb hardness that, therefore, was the lowest in the control bread and the highest in the 20% hempseed protein-enriched bread. The limited hardness in the enriched bread was maintained also during the storage, thus limiting the bread’s aging. Contrarily, in the biscuits, the volume decreased after the hempseed flour substitution, whereas the cookies hardness increased, reaching the highest value in the 60% hempseed flour enriched cookies [176]. In both bread and biscuits, the colour of crumb and cookies became darker after the hempseed product substitution as the substitution level increased; this was found to positively affect the product sensory acceptability [175,176]. Moreover, for the gluten-free bread, either the enrichment with hempseed flour or hempseed protein conferred an aroma and taste considered better than the control, nevertheless the taste acceptability was significantly reduced by 20% hempseed flour [175], whereas, as regard the biscuits, the sensory panel highlighted a significant reduction in the sensorial acceptability of the hempseed flour cookies than the control one, especially at a high level (60%) of substitution [176].

Similar results about the water absorption properties, colour, and nutritional and antioxidant changes after the addition of hempseed flour in the mixture, were obtained by Radocaj and colleagues [177] for gluten-free crackers in which brown rice flour was partially substituted with 10%, 20%, 30%, or 40% of hempseed flour. In this work, the authors underlined the possible employment of hempseed flour as a functional ingredient usable to mix with other functional raw materials such as green tea leaves, as it was done in this study, to enhance the nutritional and healthy values of daily food and to develop potentially functional foods, accessible also to the coeliac people. Indeed, with this report, the researchers demonstrated that the addition of hempseed flour is the direct responsible to some nutritional improvement that was even better than those observed for other fortified or enriched products. For example, the authors demonstrated that the addition of hempseed flour permitted to obtained crackers with a content of proteins, fibre, and minerals higher than that found for crackers enriched with pulse flour. Moreover, the addition of hemp flour led to enforcement of the crackers with essential FAs that lack in other analogous products. This remark was in line with the findings by Norajit and colleagues [178], who investigated the effects of the addition of hempseed flour, or whole hempseeds powder, at three different levels (20%, 30%, or 40%), on the nutritional quality and the antioxidant activity of rice flour bars. Interestingly, comparing the nutritional and functional differences (i.e., antioxidant activity) between the rice bars enriched with hempseed flour and that enriched with whole hempseeds powder, the authors showed that the rice bar with whole hempseeds powder had significantly higher total phenolic and flavonoid contents, as well as better antioxidant properties, than the rice bar blended with hempseed flour.

A few studies investigated the incorporation of whole hempseeds or derivatives products (i.e., de-hulled hempseed, hempseed flour, and hempseed proteins) in meat products in order to ameliorate their nutritional value. In this context, Zając and Świątek [179] used the whole hempseeds as added ingredients of liver pâtés with the aim to improve its FA profile. Actually, although the total fat content of the tested pâtés did not significantly change, its FA profile was significantly better since MUFAs and SFAs decreased compared to the control whilst the PUFAs increased. Moreover, the total n-3 PUFAs was almost 6-fold higher than the control product, and even though its level was too low to label the product as a source of n-3 PUFAs accordingly to the EU regulation 1924/2006, nevertheless, its significantly higher amount conferred it a better nutritional value in comparison with the traditional pâtés. The authors also assessed the texture and sensorial properties of the experimental product and demonstrated that the addition of whole hempseeds significantly raised the hardness, chewiness, and the adhesiveness of the pâtés without significantly changed its cohesiveness. Despite these texture variations, the inclusion of whole hempseed did not modify the sensory parameters compared to the control.

Zając and colleagues [180] also investigated the effects of the incorporation of 5% of either whole hempseeds, de-hulled hempseeds, hempseed flour, or hempseed proteins in pork loaves, on nutritional quality, sensorial acceptability, and physical features of the obtained product. From a nutritional point of view, the obtained data showed that, among all hempseed additives, only the whole hempseeds have led to a significant increase in protein, ash, and total fibre content, without significantly changing the total fat amount, whereas, hempseed flour and proteins were the additives that have led to the highest enrichment in the amounts of K, Mn, and Fe, and of Ca, Mg, and Zn, respectively, even though a significant increase in the macro- and micro-elements in comparison to the control, was observed also for the others hempseed additives, in spite of in a minor extent. Furthermore, predictably, only the inclusion of whole hempseeds and de-hulled hempseeds led to a significant improvement in the FAs profile, through decreasing the total MUFAs and SFAs, increasing the total PUFAs, both n-3 PUFAs (4-times and 3-times higher in whole and de-hulled hempseeds enriched sample, respectively) and n-6 PUFAs (2-times higher) and decreasing the n-6/n-3 PUFAs ratio. The improvement of the FA profile of the pork loaves enriched with whole or de-hulled hempseeds, led these samples to have also the lowest thrombogenicity and atherogenicity index. Hence, overall, these two enriched products appeared to be healthier and nutritionally better in comparison to the other enriched pork loaves. Nevertheless, a Thiobarbituric Acid Reactive Sunstances (TBARS) analysis showed that the de-hulling process made the pork loaves enriched with de-hulled hempseeds more prone to oxidation during the storage in comparison to the other samples, probably due to the removal of the hull, that as previously said, it has shown to be the hempseed fraction where most of polyphenols are located [74,92,100]. However, among all evaluated samples, the de-hulled hempseeds enriched one was the sample which has received the higher consumers’ acceptance, even though, interestingly, the authors found that the consumers’ willingness to buy the meat enriched with hempseed products increased after providing the information about the healthy ingredient and therefore, highlighted the importance to furnish the right awareness to consumers about the hempseed’s health benefits.

Finally, hempseed proteins have been also used as an additive for yogurt by Dabija and colleagues [181] with the aim to fortify it and to obtain a new daily product with high nutritional value. In this study, the authors found that among different tested vegetable source proteins (i.e., hempseed proteins, pea proteins, gluten wheat, soy proteins, and pumpkins flour proteins), hempseed proteins have been shown to be those with the lowest syneresis value, which is an index of the expulsion grade of serum, considered as a negative acceptable parameter. This could be related to the high fibre content of hempseed proteins that improved the viscosity and reduced the serum separation levels, as it was confirmed by the rheological analysis. Moreover, the addition of hempseed proteins also induced a reduction in the pH value and an increase in the acidity of the fortified yogurt. Both features can be related to an improvement of the growth of the bacteria present in the yogurt. In addition to these positive changes, no significant differences were found about the acceptance of the fortified sample with hempseed proteins in comparison to the control sample.

Hence, in view of the above, it is clear that there are efforts by researchers to understand the structural, nutritional, and functional roles of the hempseeds and derivative utilization in the development of new heathier and nutritionally improved foods, also for specific intended groups of people such as coeliac ones. However, further investigations are needed for a better comprehension of the most appropriate hempseed product types, matrices, and dosages in order to develop a food product with a greater improvement of nutritional and functional properties, without negatively affecting the physical and sensorial properties of the food product. In-depth investigations are also necessary to identify the most functional hempseed’s compounds, their molecular mechanisms, and their targets in order to comprehend the most adequate concentration of the functional compounds and the vehicle/matrix most suitable to preserve their structure and functionality into the gastrointestinal tract to maximize their bioavailability in the human body and to allow the compounds to reach the target sites. Only in this way, the regular ingestion of the appropriate quantity of food product could actually exert a preventive and beneficial clinically detectable effect, as required in order to claim a food as a functional food.

## 6. Conclusions

Following the recovery of the industrial hemp cultivation (i.e., *C. sativa* L. *cvs* containing a THC level <0.3 or 0.2% of dry weight of the reproductive part of the female plant at flowering), in the last decades there has been a growing interest for the edible fruits of this plant, namely hempseeds, which were initially considered as a by-product of the hemp technical fibre industry, despite their high nutritional and health beneficial power, which has started to be investigated relatively recently. The analysis of scientific literature showed that, nutritionally, these oilseeds contain 25–35% lipids with unique and perfectly balanced FA profiles, characterized by an over 80% amount of PUFAs, with the EFAs n-6 LA and n-3 ALA in the perfectly ratio as suggested for human nutrition. Moreover, the unsaponifiable oil fraction of hempseeds is rich in tocopherols with high antioxidant activity, essential to protect the PUFAs from oxidation, as well as phytosterol such as β-sitosterol. Hempseeds also contain 20–25% proteins of high biological value, easy to digest and rich in essential amino acids, and a high amount of macro- and micro-elements. In addition to their high nutritional value, hempseeds are also characterized by the presence of different bioactive compounds, among which are unique phenolics as well as bioactive peptides with antioxidant, anti-inflammatory, neuroprotective, antihypertensive, antiproliferative, and hypocholesterolemic activities, assessed mainly by in vitro studies. However, hempseed does not completely lack of some antinutritional compounds, which could negatively affect the proteins and the minerals digestion and bioavailability, above all TIs and phytic acid.

Due to the optimal nutritional composition and to the functional properties of hempseeds, some researchers investigated on one hand, the effects of hempseed dietary supplementation on health and in specific pathological condition, through animal models and humans studies and, on other hand, the possibility to used hempseeds in the food industry as livestock feed supplement to ameliorate the nutritional quality of animal-derived food products, and as ingredient to enrich or fortify daily foods. Overall, the studies performed on animal models have highlighted that there is some evidence about the protective and beneficial effects of hempseeds dietary supplementation especially on inflammatory and chronic-degenerative diseases such as CVDs and neurodegenerative ones, whereas, from the human studies some benefits for the atopic dermatitis treatment, certain blood parameters, and inflammatory responses modulation were emerged following the hempseed oil dietary intake. Nevertheless, the papers present in the scientific literature concerning this issue, are still few and rather inconclusive, referring only to hempseed oil, and is not very comparable due to the employiment of different types of experimental designs, as well as different dosages, experimental periods, and administration methods of the supplement. Finally, the studies respecting the adoption of hempseeds or derivatives in the food industries showed that these products could effectively ameliorate the FAs profile of some animal foods like eggs, milk, and meat, when they are used as livestock feed supplement, whereas when they are employed as ingredients of enriched or fortified foods, many changes occurred in both the rheological and sensorial acceptability of the enriched/fortified products and, often, they were not positively in spite of the improvement of the nutritional quality. Therefore, further investigations are needed to expand the research and knowledge about the potential use of the hempseed or derivatives for the development of functional foods. Particularly, it is necessary to understand what are the most functional hempseed’s compounds, their molecular mechanisms, and their targets, in order to comprehend the most adequate concentration of the functional compounds and the vehicle/matrix most suitable to preserve their structure and functionality into the gastrointestinal tract, and to maximize their bioavailability in the human body. Furthermore, it is also required to identify the best compromise between the functionality and the rheological and sensorial properties, taking into account that a certain reduction of sensorial acceptability could be tolerable by consumers informed about the beneficial effects of the fortified/enriched products in comparison to the common counterpart.

## Figures and Tables

**Table 1:** Industrial hemp *cultivars* registered and listed in the European Union Plant Variety Database and their origin. All these varieties are certified to contain less than 0.2% of THC of dry weight of leaves and flowering parts of the plant, according to the EU regulation 1307/2013.

No	Registered Varieties	Origin	No	Registered Varieties	Origin
1	Adzelvieši	Latvia	33	Ivory	Nederland
2	Armanca	Romania	34	KC Bonusz	Hungary
3	Asso	Italy	35	KC Dora	Hungary
4	Austa SK	Latvia	36	KC Virtus	Hungary
5	Balaton	Hungary	37	KC Zuzana	Hungary
6	Beniko	Switzerland	38	KCA Borana	Hungary
		Czech Republic			
		Nederland			
		Poland			
7	Bialobrzeskie	Czech Republic Polan	39	Kompoliti	Hungary
8	Cannakomp	Hungary	40	Kompoliti Hibrid TC	Hungary
9	Carma	Italy	41	Lipko	Hungary
10	Carmaleonte	Italy	42	Lovrin 110	Romania
11	Chamaeleon	Nederland	43	Marcello	Nederland
12	Codimono	Italy	44	Markant	Nederland
13	Dacia Secuieni	Romania	45	Monoica	Switzerland
					Hungary
14	Delta-405	Spanish	46	Orion 33	France
15	Delta-Ilosa	Spanish	47	Rajan	Poland
16	Dioica 88	France	48	Ratza	Romania
17	Earlina 8 FC	France	49	Santhica 23	France
18	Eletta Campana	Italy	50	Santhica 27	France
19	Epsilon 68	France	51	Santhica 70	France
20	Fedora 17	Switzerland	52	Secuieni Jubileu	Romania
		France			
21	Felina 32	France	53	Silvana	Romania
22	Fibrante	Italy	54	Succesiv	Romania
23	Fibrol	Hungary	55	Teodora	Romania
24	Fibror 79	France	56	Tiborszallasi	HungaryItaly
25	Finola	Finland	57	Tisza	Hungary
26	Futura 75	France	58	Tygra	Poland
27	Futura 83	France	59	Uniko B	Hungary
28	Fèrimon	Deutschland France	60	Uso-31	Nederland
29	Glecia	Italy	61	Villanova	Italy
30	Gilana	Italy	62	Wielkopolskie	Poland
31	Glyana	Poland	63	Wojko	Poland
32	Henola	Poland	64	Zenit	Romania

**Table 2:** Hempseed nutritional characteristics (mg/100 g).

Moisture	Fat	Proteins	CHO	Total DF	Insoluble DF	Soluble DF	Ash	Ref
1.1–7.2	26.9–30.6	23.8–28.0	n.a.	n.a.	n.a.	n.a.	5.1–5.8	[34]
4.1–4.3	32.8–35.9	24.3–28.1	32.5–37.5	n.a.	n.a.	n.a.	4.9–6.1	[44]
6.7 ± 0.5	34.6 ± 1.2	25.6 ± 0.6	34.4 ± 1.5	33.8 ± 1.9	30.9 ± 1.5	2.9 ± 0.4	5.4 ± 0.3	[45]
4.0–9.2	25.4–33.0	21.3–27.5	n.a.	n.a.	n.a.	n.a.	3.7–5.9	[46]
6.5	35.5	24.8	27.6	27.6	22.2	5.4	5.6	[47]
8.4 ± 0.02	33.3 ± 0.1	22.5 ± 0.2	n.a.	n.a.	n.a.	n.a.	5.9 ± 0.03	[48]
7.3 ± 0.1	24.5 ± 2.0	24.8 ± 1.1	38.1 ± 2.5	n.a.	n.a.	n.a.	5.3 ± 0.6	[49]

Where more than one *cultivar* has been analysed, the maximum and minimum values of the obtained data range are shown. Where only one *cultivar* has been analysed, the mean ± standard deviation is reported. n.a.: not available; CHO: carbohydrates; DF: Dietary Fibre.

**Table 3:** Hempseed fatty acid profile (% of oil).

PA	SA	OA	LA	GLA	ALA	SDA	∑ SFA	∑ MUFA	∑ PUFA	n-6/n-3	Ref.
6.7–7.0	2.1–2.8	9.4–13.0	55.6–56.6	2.6–4.5	14.7–17.3	n.a.	9.6–10.3	n.a.	n.a.	n.a.	[34]
6.0–7.3	2.3–3.5	9.2–15.7	55.0–58.2	0.6–4.5	12.6–19.6	0.2–1.5	9.4–11.7	9.7–16.1	72.2–80.7	2.8–4.5	[39]
7.1–9.1	2.1–2.8	10.3–17.9	51.6–54.2	1.9–5.0	10.5–15.3	n.a.	n.a.	n.a.	n.a.	3.9–5.5	[3]
6.1–7.8	2.3–4.0	12.2–18.8	53.9–59.0	3.5–6.2	12.3–18.9	n.a.	n.a.	n.a.	72.0–78.6	3.2–5.0	[44]
5.0	2.0	9.0	56.0	4.0	22.0	2	n.a.	n.a.	84.0	2.5	[47]
7.0 ± 0.3	2.8 ± 0.4	12.7 ± 1.3	56.2 ± 3.5	2.9 ± 0.4	15.0 ± 1.1	n.a.	10.9 ± 0.5	13.1 ± 1.3	75.1 ± 3.7	4.0	[49]
5.6 ± 0.5	3.9 ± 0.4	16.2 ± 6.2	54.7 ± 4.1	n.a.	16.2 ± 4.0	0.5 ± 0.01	10.9 ± 0.7	17.5 ± 6.3	72 ± 4.3	3.2	[50]

Where more than one *cultivar* has been analysed, the maximum and minimum values of the obtained data range are shown. Where only one *cultivar* has been analysed, the mean ± standard deviation is reported. PA: Palmitic Acid (16:0); SA: Stearic Acid (18:0); OA: Oleic Acid (18:2, n-9); LA: Linoleic Acid (18:2, n-6); GLA: γ-Linolenic Acid (18:3, n-6); ALA: α-Linolenic Acid (18:3, n-3); SDA: Stearidonic Acid (18:4, n-3); ∑-SFA: Total Saturated Fatty Acid; ∑-MUFA: Total Monounsaturated Fatty Acid; ∑-PUFA: Total Polyunsaturated Fatty Acid; n.a.: not available.

**Table 4:** Hempseed oil unsaponifiable matter: tocopherol profile.

Type of Analyzed Sample	Total Tocopherols	α-Tocopherol	β-Tocopherol	γ-Tocopherol	δ-Tocopherol	Ref.
mg/100 g seed						
Whole seed	n.a.	n.a.	n.a.	21.21–294.9	62.0–115.7	[34]
Oil	14.33–34.04	0.70–3.05	0.06–0.30	15.42–29.40	0.51–2.49	[41]
Whole seed	n.a.	n.a.	n.a.	4.6–11.3	0.6–1.3	[3]
Whole seed	90	5	n.a.	85	n.a.	[47]
Whole seed	n.a.	0.35 ± 0.09	n.a.	0.55 ± 0.13	n.a.	[49]
mg/100 g oil						
Whole seed	61–135	n.a.	n.a.	n.a.	n.a.	[39]
Oil	56.28–58.22	23.4–24.62	31.6–32.20 ^1^		1.28–1.40	[48]
Oil	n.a.	2.78 ± 0.01	n.a.	56.41 ± 0.02	n.a.	[52]
Oil	80.28 ± 4.50	3.22 ± 0.65	0.81 ± 0.16	73.38 ± 2.86	2.87 ± 0.83	[57]
Oil	n.a.	2.56 ± 0.06	0.60 ± 0.005	59.79 ± 1.21	3.97 ± 0.15	[58]
Oil	79.7	3.4	0.6	73.3	2.5	[59]
Oil	97.13	4.32 ± 0.65	n.a.	89.26 ± 1.92	3.55 ± 0.25	[60]
Oil	92.97	4.71 ± 0.54	n.a.	84.11 ± 1.74	4.15 ± 0.44	[61]

^1^ β-tocopherol + γ-tocopherol. Where more than one *cultivar* has been analysed, the maximum and minimum values of the obtained data range are shown. Where only one *cultivar* has been analysed, the mean ± standard deviation is reported. n.a.: not available.

**Table 5:** Hempseed oil unsaponifiable matter: phytosterol profile.

Type of Analysed Sample	Total Phytosterols	β-Sitosterol	Stigmasterol	Campesterol	Ref.
mg/100 g seed					
Whole seed	n.a.	53.61 ± 3.15	2.47 ± 0.25	11.54 ± 1.03	[49]
Whole seed	124 ± 12	79.7 ± 0.1	3.4 ± 0.9	7.3 ± 0.6	[50]
mg/100 g oil					
Oil	279.37 ± 12.43	190.5 ± 5.93	10.02 ± 0.75	50.57 ± 3.20	[57]

Data are expressed as mean ± standard deviation. n.a.: not available.

**Table 6:** Hempseed protein amino acid content (g/100 g seed).

Ala	Arg	Asp	Cys	Glu	Gly	His *	Ile *	Leu *	Lys *	Met *	Phe *	Pro	Ser	Thr *	Trp *	Tyr	Val *	Ref.
1.28	3.10	2.78	0.41	4.57	1.14	0.71	0.98	1.72	1.03	0.58	1.17	1.15	1.27	0.88	0.20	0.86	1.28	[47]
1.23	2.76	2.13	0.31	4.58	1.24	0.64	0.62	1.53	1.28	0.50	1.02	1.14	1.27	0.85	n.a.	0.73	0.68	[48]
0.94	2.69	2.33	0.36	3.83	1.02	0.58	0.86	1.47	0.84	0.56	1.01	0.89	1.12	0.79	n.a.	0.78	1.13	[45]
0.96	2.28	2.39	0.41	3.74	1.06	0.55	0.80	1.49	0.86	0.56	1.03	0.90	1.19	1.01	0.23	0.68	1.14	[46]

* Essential amino acids. Ala: alanine; Arg: arginine; Asp: asparagine; Cys: cysteine; Glu: glutamate/glutamine; His: histidine; Iso: isoleucine; Leu: leucine; Lys: lysine; Met: methionine; Phe: phenylalanine; Pro: proline; Ser: serine; Thr: threonine; Trp: tryptophan; Tyr: tyrosine; Val: valine; n.a.: not available.

**Table 7:** Hempseed mineral composition value (mg/100 g) of different industrial hemp *cultivars*.

P ^1^	K ^1^	Mg ^1^	Ca ^1^	Na ^1^	Fe ^2^	Mn ^2^	Zn ^2^	Cu ^2^	Cd ^2^	Ref.
1160	859	483	145	12	14	7	7	2	n.a.	[47]
890	n.a.	240	90	n.a.	7	6	6	n.a.	n.a.	[48]
n.a.	252	268	94	6.8	10	4	5	0.5	n.a.	[49]
n.a.	463–2821	237–694	144–955	n.a.	113–240	6–11	4–9	n.a.	0.1–0.4	[43]
1014–910	727–866	430–482	94–121	22–27	11–13	12–15	10–11	0.8–0.9	n.a.	[44]
1170	921	496	127	n.a.	4	11	7	1.9	0.0015	[45]

^1^ Macro-element. ^2^ In-trace element. Where more than one *cultivar* has been analysed, the maximum and minimum values of the obtained data range are shown. Where only one *cultivar* has been analysed, the mean ± standard deviation is reported. P: phosphorous; K: potassium; Mg: magnesium; Ca: calcium; Na: sodium; Fe: iron; Mn: manganese; Zn: zinc; Cu: copper; Cd: cadmium. n.a.: not available.

**Table 8:** Hempseed antinutritional compounds.

Phytic Acid ^1^	Condensed Tannins ^2^	Trypsin Inhibitors ^3^	Cyanogenic Glycosides ^4^	Sapoins ^2^	Ref.
4.5–7.6 *	n.a.	10.8–27.8 *	n.a.	n.a.	[39]
6.2–7.7 *	214.0–456.0 *	10.8–27.7 *	50.0–170.0 *	47.0–70.0 *	[73]
5.3 ± 0.4 *	105.0 ± 4.0 **	n.a.	n.a.	n.a.	[74]
6.1–7.4 *	135.0–214.0 *	18.2–28.4 *	60.0–240.0 *	5.9–8.1 *	[75]

^1^ g/100 g. ^2^ mg/100 g. ^3^ U/mg. ^4^ ppm. * On defatted matter. ** On whole seed. Where more than one *cultivar* has been analysed, the maximum and minimum values of the obtained data range are shown. Where only one *cultivar* has been analysed, the mean ± standard deviation is reported. n.a.: not available.

**Table 9:** Total phenolic content (mg Gallic Acid Equivalent (GAE)/g) of hempseed cold-pressed oil or whole hempseed.

Type of Sample	TPC	Ref.
Whole hempseed	13.68–51.60	[34]
Whole hempseed	3.82–7.80	[3]
Whole hempseed	0.77 ± 0.04	[49]
Cold-pressed oil	1.88 ± 0.003	[52]
Whole hempseed	0.96 ± 0.35	[74]
Cold-pressed oil	2.68 ± 0.09	[95]
Cold-pressed oil	0.44 ± 0.001	[96]
Whole hempseed	0.92 ± 0.04	[98]
Whole hempseed	2.33 ± 0.07	[99]

Where more than one *cultivar* has been analysed, the maximum and minimum values of the obtained data range are shown. Where only one *cultivar* has been analysed, the mean ± standard deviation is reported. TPC: Total Phenolic Content.

**Table 10:** Hempseed phenylpropionamides and their biological effect.

Compound	Biological Effect	Ref.
*N-trans-*caffeolyltyramine	Strong in vitro scavenging DPPH radical and ORAC antioxidant activity; LDL protection against in vitro oxidation; In vitro AChE and arginase inhibitory activity; In vivo anti-neuroinflammatory action; In vitro prevention of H_2_O_2_-induced cell death.	[100] [103] [104] [107] [111]
*N-trans-*feryoryltyramine	In vitro scavenging DPPH radical activity; In vivo anti-neuroinflammatory action.	[103] [111]
*N-trans-*caffeolyoctopamine	In vivo anti-neuroinflammatory action.	[111]
*N-trans-*cumaroyltyramine	In vivo anti-neuroinflammatory action.	[111]
Cannabisin A	In vitro scavenging DPPH radical activity; In vivo anti-neuroinflammatory action.	[103] [111]
Cannabisin B	Strong in vitro scavenging DPPH radical and ORAC antioxidant activity; LDL protection against in vitro oxidation; In vitro arginase inhibitory activity.	[100] [104] [111]
Cannabisin C	In vitro scavenging DPPH radical activity; In vivo anti-neuroinflammatory action.	[103] [111]
Cannabisin D	In vitro scavenging DPPH radical activity; In vivo anti-neuroinflammatory action.	[103] [111]
Cannabisin E	In vivo anti-neuroinflammatory action	[111]
Cannabisin F	In vitro scavenging DPPH radical and ORAC antioxidant activity; In vivo anti-neuroinflammatory action; In vitro inhibition of the NFκB inflammatory pathway, activation of Nfr-2 antioxidant pathway and upregulation of SIRT1.	[104] [110] [111]
Cannabisin M	In vitro scavenging DPPH radical activity; In vivo anti-neuroinflammatory action.	[103] [111]
Isocannabisin N	In vivo anti-neuroinflammatory action	[111]
Grossamide	In vitro inhibition of the NFκB inflammatory pathway. In vivo anti-neuroinflammatory action;	[109] [111]
3,3′-demethyl-grossamide	In vitro scavenging DPPH radical activity; In vitro AChE inhibitory activity; In vivo anti-neuroinflammatory action.	[103] [111]
3,3′-demethylheliotropamide	In vitro AChE and arginase inhibitory activity.	[103]
Sativamides A	In vitro reduction of ER stress-induced cell death.	[106]
Sativamides B	In vitro reduction of ER stress-induced cell death.	[106]
4-((E)-p-coumaroylamino)butan-1-ol	In vitro inhibition of TNF-α release.	[105]
Cumaroylaminobutanol glucopyranoside	In vitro inhibition of TNF-α release; In vitro inhibition of the NFκB inflammatory pathway and activation of Nfr-2 antioxidant pathway.	[105] [108]

**Table 11:** Literature studies on the effect of hempseed or hempseed products’ dietary supplementation on animal models.

Supplement Type	Purpose	Experimental Design	Results	Ref.
Hempseed meal hydrolysate (HMH)	Evaluation of the antioxidant ability of an HMH-containing diet to attenuate the plasma levels of some oxidative stress markers in growing and adult SHRs rats.	8-week old SHRs (*n* = 8) fed control or 1% HMH-containing diets *ad libitum* for 8 weeks (for analysing the preventive effect). 20-week old SHRs (*n* = 4) fed control or HPH-containing diets *ad libitum* for 4 weeks (for analysing the treatment effect).	Dietary intervention with a specific HMH on young and adults SHRs exerted an antioxidant action by: • The increase of the SOD and CAT activities in both growing and adult SHRs; • The significant decrease of the TPx plasma level in both growing and adult SHRs and NTRs.	[117]
Hempseed	Investigation of the aspects of dietary hempseed against hyperlipidemia-associated CV risks.	1 month of 10% hempseed supplemented high fat-diet on Wistar rats.	The 10% hempseed dietary treatment had an anti-hyperlipidaemic effect by inducing: • The serum level decreases of the cholesterol, LDL and triglycerides; • The increase of the HDL serum level; • A significant reduction in the aorta thickness; • Less intima damage foam cells formation and smooth muscle cell proliferation; • The remarkable decrease in the lipid deposition, plaque size, and lesion surface area distribution; • The decrease of the pro-inflammatory PGEs expression; • The increase of the anti-inflammatory PGDs expression.	[131]
Hempseed	Evaluation of the effect of hempseed feeding on lipid and protein profiles.	20-days of hempseed supplementation (free access) on Wistar rats.	Short-term hempseed dietary intake can have hepatoprotective properties and beneficial effect on the prevention of CVD by favouring: • The decrease of the mean fasting serum LDL level; • The increase of the mean fasting serum HDL and total protein levels.	[132]
Hempseed water extract (HWE)	Evaluation of the anti-atherosclerotic activity of hempseed water extract in ApoE KO mice.	14-days of intragastric inoculation of 300 µL of hempseed water extract or distilled water (control) to six-week-old, male ApoE KO (*n* = 7).	Dietary HWE ingestion exerted an anti-atherosclerotic effect through: • The reduction of the atherosclerotic plaque formation in the aortic sinuses; • The reduction of the serum GOT concentration, demonstrating less liver damage after the treatment; • The decrease of total serum cholesterol and LDL, and the increase of serum HDL; • The significant decrease of TNF-α serum level.	[133]
Hempseed Meal (HSM)	Investigation of the protective effects of the dietary intake of HSM and its LA and ALA components on AD and CVD *Drosophila melanogaster* models.	GMR-Aβ42 *Drosophila melanogaster* line (AD model) reared in cornmeal-soybean standard media with the LA, ALA, and GLA amounts present in the HSM (for the effect on AD phenotype). Wild-type *Drosophila melanogaster* larvae reared in HSM media with 35.1 μg/mL of cholesterol (for the CV effect).	Thanks to their LA and ALA contents, HSM dietary uptake may exert beneficial effects on AD and CVD through: • The suppression of suppressed Aβ42 cytotoxicity in the GMR-Aβ42 flies’ line reared on HSM mean (beneficial effect on AD); • The reduction of dietary cholesterol uptake observed in the wild-type flies reared on the cholesterol media (beneficial effect on CVD).	[134]
Hempseed	Investigation of the ability of dietary hempseed to reduce platelet aggregation under hypercholesterolemia.	Male New Zealand rabbits fed 125 g/day of control diet supplemented with 10% of hempseed and 0.5% of cholesterol for 8 weeks.	Hempseed supplementation to a hypercholesterolemic diet is able to normalize the platelet aggregation by increasing the GLA plasma level.	[135]
Hempseed	Investigation of the dietary hempseed ability to inhibit atherosclerosis and the associated vascular contractile dysfunction in a cholesterol-induced rabbit model.	Male albino New Zealand rabbits fed 125 g/day of control diet supplemented with 10% of hempseed and 0.5% of cholesterol (to induce atherosclerotic plaque formation) for 8 weeks.	Dietary hempseed supplementation did not generate protection against atherosclerotic plaque formation in hypercholesterolemic diet, but it provided mildly beneficial effects against contractile dysfunction associated with atherosclerotic vessels.	[136]
Hempseed	Investigation of the ability of hempseed dietary intake on the prevention of ovarian hormone deficiency-induced hypercholesterolemia in ovariectomized rats and on the complications of estrogen deficiency such as depression, anxiety, and bone loss problems.	Bilateral ovariectomized six-month-old female Wistar rats (*n* = 5) fed 1%, 2%, or 10% hempseed supplemented diets or a control diet (standard commercial diet) for 3 weeks.	Until 10% of hempseed dietary supplementation improved the post-ovariectomized complications in rats since it: • Prevented the accrual of blood calcium, preventing the bone loss; • Prevented the body weight gain induced by ovariectomy; • Conserved the TGs and TC plasma levels as those of the non-ovariectomized rats, preventing dyslipidaemia; • Resulted in an antidepressant activity.	[137]
Hempseed meal hydrolysate (HMH)	Evaluation of the effect of HMH-containing diet on the prevention and treatment of hypertension in growing SHRs and in SHRs with established hypertension.	8-week old SHRs (*n* = 8) fed control or 1% HMH-containing diets *ad libitum* for 8 weeks (for the preventive effect). 20-week old SHRs (*n* = 4) fed control (without HPH) or HPH-containing diets, *ad libitum* for 4 weeks (for analysing the treatment effect).	Dietary intervention with a specific HMH on young and adult SHRs exerted a preventive and therapeutic effects since it led to: • The attenuation of the normal increases of SBP in young growing SHRs; • The significant reduction of SBP in adult SHRs; • The significant reduction of the plasma ACE and renin activities in both young and adult SHRs.	[138]
Hempseed and bitter vegetable (HB)	Evaluation of the beneficial effect of the HB diet on chronic diseases and on the slowing of the aging process.	15-month-old C57BL/6 female mice (*n* = 10) fed low CHO, low SFA, and high PUFA content-HB diets containing hempseed and bitter vegetable (*Sonchus oleraceus*) (2:1) or control (western diet), *ad libitum* until only 20% of the mice in one group survived.	The HB diet is capable of promoting health and longevity since it: • Significantly increased the mice lifespan; • Improved the spatial learning and memory, and the locomotory activity; • Exerted beneficial effects on hepatic steatosis, hepatotoxicity, and plasma lipid profiles including the normalization of the size, weight, and colour of the liver and the reduction of hepatic lipid accumulation and of the ALT, AST, and TG levels. • Provided protective effects against the aging-related abnormalities in spleen morphology and inflammation; • Increased the antioxidant defences and decreased the oxidative stress; • Decreased the systemic inflammation by lowering the plasma level of the inflammatory cytokines (i.e., TNF-α, IL-1β, MCP-1 and IL-6), and by increasing the plasma level of anti-inflammatory IL-10; • Improved the gut microbiota profile leading to a significant decrease in the gut levels of *E. coli* and a significant increase in the gut levels of *Bifidobacterium* and *Lactobacillum* species after 11 weeks of dietary intervention; • Increased the sensitivity to insulin; • Increased the hepatic expression of genes associated with longevity (i.e., AMPK, Sirt1, Nrf-1, and FOXO3).	[140]
Hempseed oil and Evening primrose oil (HSO/EPO)	Evaluation of the MS’ therapeutic effect of co-supplementation with HSO/EPO.	14 days of HSO/EPO oral administration on adult female C57BL/6 (*n* = 6) EAE mice (MS model)	The HSO/EPO dietary treatment can exert a beneficial effect on MS treatment since it is able to: • Act as an immunomodulator, down-regulating the mRNA expression of mTORC1 (which promotes Th1 and Th2 cells differentiation) and of the pro-inflammatory cytokines IFN- in lymph node; • Exert an anti-inflammatory action through increasing the mRNA expression of mTORC2 (which promotes Th2 cells differentiation) and of the anti-inflammatory cytokines IL-10 in lymph nodes; • Reduce the infiltration of the inflammatory cells in brain; • Promote the re-myelination of neurons’ myelin sheath.	[140,141]

ACE: angiotensin I-converting enzyme; AD: Alzheimer Disease; ALA: α-Linolenic Acid; ALT: Alanine Transaminase; AMPK: AMP-activated protein kinase; ApoE KO: Apolipoprotein E Knockout; AST: Aspartate Aminotransferase; CAT: Catalase; CHO: Carbohydrate; CVD: Cardiovascular Disease; EAE: Experimental Autoimmune Encephalomyelitis; FOXO3: Forkhead box O3; GLA: γ- linolenic acid; GOT: glutamic oxaloacetic transaminase; HDL: High Density Lipoprotein; IFN-γ: Interferon γ; LA: Linoleic Acid; LDL: Low Density Lipoprotein; MCP-1: Monocyte Chemoattractant Protein 1; MS: Multiple Sclerosis; mTORC1: mTOR Complex 1; mTORC2: mTOR Complex 2; rf-1: Nuclear respiratory factor 1; NTRs: Normotensive Rats; PGD: Prostaglandin D; PGE: Prostaglandin E; PUFA: Polyunsaturated Fatty Acid; SBP: Systolic Blood Pressure; SFA: Saturated Fatty Acid; SHRs: Spontaneously Hypertensive Rats; Sirt1: Sirtuin 1; SOD: Superoxide dismutase; TC: Total Cholesterol; TG: Triglyceride; IL: Interleukin; TGs: Triglycerides; TNF-α: Tumor Necrosis Factor α; TPx: Total Peroxide.

**Table 12:** Literature studies on the effect of hempseed oil dietary supplementation on humans.

Supplement Type	Purpose	Experimental Design	Results	Ref.
Cold-pressed hempseed oil	Evaluation of the effect of dietary hempseed oil and olive oil on plasma lipid profiles, TEWL, skin quality, and dermal medication usage in patients with atopic dermatitis.	Controlled, randomized single-blind crossover study, based on two intervention periods of 8-weeks last, separated by a 4-weeks washout period. Patients (*n* = 20) with a diagnosis of atopic dermatitis were randomly divided into two groups. Every patient of each group orally consumed 30 mL/day of olive or hempseed oils. • Inclusion criteria: BMI < 30 Kg/m^2^; 25–60 years old. • Exclusion criteria: assumption of lipid-lowering, anti-hypertensive or asthma medications, nutrient supplements, steroid-containing skin creams, oral cyclosporine, and use of solarium during the study and in the previous month.	A relatively short period of hempseed oil dietary consumption significantly improved skin quality and the atopic symptomology of the patients without any negative side effects or adverse reactions. The dietary treatment significantly increased the plasma level of LA, ALA, and GLA; significantly decreased the TEWL values and patients’ skin dryness, itchiness, and dermal medication usage.	[143]
Cold-pressed under nitrogen atmosphere hempseed oil	Evaluation and comparison of the effects of dietary hempseed oil and flaxseed oil on the profile of serum lipids, fasting serum total and lipoprotein lipid concentrations, plasma glucose and insulin concentrations, and hemostatic factors in healthy volunteers.	Controlled, randomized single-blind crossover study, based on two intervention periods of 4-weeks last, separated by a 4-weeks washout period. Male (*n* = 8) and female (*n* = 6) healthy volunteers were randomly divided into two groups. Every volunteer of each group orally consumed 30 mL/day of flax or hempseed oils. • Inclusion criteria: BMI < 30 Kg/m^2^; 25–60 years old; fasting serum concentration of TG < 3.5 mmol/L; fasting serum concentration of TC 5.0–7.5 mmol/L; fasting plasma glucose < 6.0 mmol/L. • Exclusion criteria: assumption of lipid-lowering, anti-hypertensive medications and nutrient supplements during the study and in the previous month.	In healthy subjects, 4-weeks daily of orally ingestion of hempseed oil: • Reduced the total plasma TGs concentration; • Significantly decreased the TC/HDL-C ratio, lowering the risk of coronary heart diseases; • Significantly changed the FA composition of serum TG and CE, increasing the LA, ALA, and GLA amounts and decreasing the OA content, whereas the AA and DHA amount did not differ; • Did not modify the plasma glucose, insulin, and hemostatic factor like fibrinogen, FVIIa, and PAI-1.	[144]
Hempseed oil capsule	Evaluation and comparison of the effects of the hempseed, flaxseed, and fish dietary oils on cardiovascular parameters (lipid profile, LDL oxidation, inflammatory markers and platelet aggregation) in healthy volunteers.	Double-blinded, placebo controlled clinical trial. Male (*n* = 34) and female (*n* = 54) healthy volunteers were randomly divided into 4 groups (placebo, fish oil, flaxseed oil, and hempseed oil). The volunteers of each group were orally daily supplemented with 2 capsules each containing 1 g of the relative treatment for 12 weeks. • Inclusion criteria: fasting TC levels <5.2 mM; non-smokers; consumption of fish not more than once a week and not more than 29.6 mL/day of alcohol consumption. • Exclusion criteria: usage of aspirin; ibuprofen; and/or other non-steroidal, anti-inflammatory medications as well as thyroid, anticoagulant, or lipid-lowering medication and the presence of menopause or chronic illness.	2 × 1g hempseed oil capsule daily supplementation, administrated for 12 weeks did not significantly affect the ALA and LA plasma levels; TC, TG, LDL, and HDL amounts, or inflammatory (CRP, and TNF-α) and platelet aggregation analyzed markers (collagen and thrombin-induced platelet aggregation).	[145]
Hempseed oil soft capsule	Evaluation of the role of hempseed oil in the modulation of hyperlipidaemia and CVD risk in children and adolescents.	Randomized, controlled, two-arm parallel-group study. Children and adolescents (*n* = 36) with diagnosis of primary hyperlipidemia were randomly divided into two groups. Each subject of the test group was dietary supplemented with one hempseed oil soft capsule containing 3 g of oil for 8 weeks. The other group was used as control (without control). • Inclusion criteria: assessment of primary hyperlipidaemia; 6–16 years old. • Exclusion criteria: diagnosis of secondary hyperlipidaemia, and/or renal, endocrine, lipid, neurologic, onco-hematologic disorders requiring drug treatment; BMI > 85th percentile; smokers.	The 8-weeks daily intake of 3 g of hempseed oil: • Did not affect the levels of TC, LDL-C, HDL-C and TGs; • Did not change the values of body weight, BMI, and blood pressure; • Significantly modified the RBC FAs composition increasing the total n-3 PUFAs, n-3 LCPUFAs, and n-6 LCPUFAs, and decreasing the n-6/n-3 PUFAs ratio as well as the SFAs and MUFAs.	[146]
Hempseed oil and evening primrose oil (HSO/EPO, 9:1) co-supplementation	Evaluation of the therapeutic and protective effect of the Hot-natured diet co-supplemented with HSO/EPO or of the HSO/EPO dietary co-supplementation on MS patients.	Double blind, randomized trial. Female (*n* = 42) and male (*n* = 23) patients with diagnosis of RMSS were randomly divided into three groups. Each group (A, B, and C) was subjected to a Hot-natured diet co-supplemented with 18–21 g of HSO/EPO (9:1) (group A) or to 18–21 g olive oil supplementation (group B), or to 18–21 g of HSE/EPO (9:1) co-supplementation (group C) for 6 months. • Inclusion criteria: diagnosis of RRMS (EDSS < 6); 14–55 years old. • Exclusion criteria: diagnosis of secondary or primary progressive MS; pregnancy; corticosteroid treatment; and diagnosis of other chronic neurological and inflammatory diseases such as cancer, rheumatic diseases, and heart diseases.	6 months of Hot-natured diet co-supplemented with HSO/EPO or of HSO/EPO co-supplementation significantly improved the MS clinical condition and inflammatory status of the patients, without any adverse effects. • Both treatments significantly improved the degree of Th2/Th1 ratio and the value of EDSS and of relapse rate; • Both treatments significantly increased the level of anti-inflammatory IL-4 cytokine; • Only the Hot-natured diet co-supplemented with HSO/EPO significantly decreased the level of pro-inflammatory IFN-γ and IL-17 cytokines; • The co-supplementation of HSO/EPO led to a decreasing (but not statistically significant) trend in the level of IFN-γ and IL-17 cytokines.	[147]
Hempseed oil and evening primrose oil (HSO/EPO, 9:1) co-supplementation	Evaluation of the Hot-natured diet co-supplemented with HSO/EPO in patients with RRMS on the type and level of produced cytokines.	Females (*n* = 16) and males (*n* = 7) patients with diagnosis of RRMS were subjected to a Hot-natured dietary regimen co-supplemented with 18–21 g of HSE/EPO (9:1), for 6 months. • Inclusion criteria: diagnosis of RRMS (EDSS < 6); 14–55 years old. • Exclusion criteria: diagnosis of secondary or primary progressive MS; pregnancy; corticosteroid treatment; and diagnosis of other chronic neurological and inflammatory diseases such as cancer, rheumatic diseases, and heart diseases.	6 months of Hot-natured diet co-supplemented with HSO/EPO had a beneficial effect in RRMS patients by decreasing the Th1 cells and by promoting the Th2 cell responses. This dietary treatment led to: • A significant improvement of EDSS; • A significant decrease of the production of the pro-inflammatory cytokines (IFN-γ and IL-17); •A significant increase of the production of anti-inflammatory IL-4.	[148]
Hempseed oil and evening primrose oil (HSO/EPO, 9:1) co-supplementation	Investigation of the effect of the Hot-natured diet co-supplemented with HSO/EPO or of the solely HSO/EPO dietary co-supplementation on MS patient’s liver dysfunction.	Double blind, randomized trial. Female (*n* = 42) and male (*n* = 23) patients with diagnosis of RMSS were randomly divided into three groups. Each group (A, B, and C) was subjected to a Hot-natured diet co-supplemented with 18–21 g of HSO/EPO (9:1) (group A) or to 18–21 g olive oil supplementation (group B), or to 18-21 g of HSE/EPO (9:1) co-supplementation (group C) for 6 months. • Inclusion criteria: diagnosis of RRMS (EDSS < 6); 14–55 years old. • Exclusion criteria: diagnosis of secondary or primary progressive MS; pregnancy; corticosteroid treatment; and diagnosis of other chronic neurological and inflammatory diseases such as cancer, rheumatic diseases, and heart diseases.	6 months of Hot-natured diet co-supplemented with HSO/EPO or of HSO/EPO co-supplementation significantly improved the MS patients’ liver dysfunction, without any adverse effects. • Both treatments significantly improved the EDSS value; • Both treatments significantly decrease the AST enzyme serum level; • Only the Hot-natured diet co-supplemented with HSO/EPO significantly decreased the ALT and GGT enzyme serum levels.	[149]

ALA: α-linolenic Acid; ALT: (SGPT) alanine-aminotransferase; AST: (SGOT) aspartate- aminotransferase; BMI: Body Mass Index; CVD: Cardiovascular Disease; EDSS: Extended Disability Status Score; FA: Fatty Acid; Cholesteryl ester; FVIIa: coagulation Factor VIIa; GGT: gamma-glutamyl transferase; GLA: γ-Linolenic Acid; HDL-C: High Density Lipoprotein-Cholesterol; LA: Linoleic Acid; LCPUFAs: Long Chain Polyunsaturated Fatty Acids; LDL-C: Low Density Lipoprotein-Cholesterol; MS: Multiple Sclerosis; MUFAs: Monounsaturated Fatty Acids; OA: Oleic Acid; PAI-1: Plasminogen activator inhibitor-1; PUFAs: Polyunsaturated Fatty Acids; RBC: Red Blood Cell; RRMS: Relapsing-Remitting Multiple Sclerosis; SDA: Stearidonic Acid; SFAs: Saturated Fatty Acids; TC: Total Cholesterol; TEWEL: trans-epidermal water loss; TG: Triglyceride.
